# Interference of Arabidopsis *N-Acetylglucosamine-1-P Uridylyltransferase* Expression Impairs Protein N-Glycosylation and Induces ABA-Mediated Salt Sensitivity During Seed Germination and Early Seedling Development

**DOI:** 10.3389/fpls.2022.903272

**Published:** 2022-06-07

**Authors:** Ya-Huei Chen, Hwei-Ling Shen, Shu-Jen Chou, Yasushi Sato, Wan-Hsing Cheng

**Affiliations:** ^1^National Defense Medical Center, Graduate Institute of Life Sciences, Taipei, Taiwan; ^2^Institute of Plant and Microbial Biology, Academia Sinica, Taipei, Taiwan; ^3^Biology and Environmental Science, Graduate School of Science and Engineering, Ehime University, Matsuyama, Japan

**Keywords:** N-acetylglucosamine, hexosamine biosynthesis pathway, protein glycosylation, unfolded protein response, ABA signaling, salt stress, N-acetylglucosamine-1-P uridylyltransferase

## Abstract

N-acetylglucosamine (GlcNAc) is the fundamental amino sugar moiety that is essential for protein glycosylation. UDP-GlcNAc, an active form of GlcNAc, is synthesized through the hexosamine biosynthetic pathway (HBP). Arabidopsis N-acetylglucosamine-1-P uridylyltransferases (GlcNAc1pUTs), encoded by *GlcNA.UT*s, catalyze the last step in the HBP pathway, but their biochemical and molecular functions are less clear. In this study, the *GlcNA.UT1* expression was knocked down by the double-stranded RNA interference (dsRNAi) in the *glcna.ut2* null mutant background. The RNAi transgenic plants, which are referred to as iU1, displayed the reduced UDP-GlcNAc biosynthesis, altered protein N-glycosylation and induced an unfolded protein response under salt-stressed conditions. Moreover, the iU1 transgenic plants displayed sterility and salt hypersensitivity, including delay of both seed germination and early seedling establishment, which is associated with the induction of ABA biosynthesis and signaling. These salt hypersensitive phenotypes can be rescued by exogenous fluridone, an inhibitor of ABA biosynthesis, and by introducing an ABA-deficient mutant allele *nced3* into iU1 transgenic plants. Transcriptomic analyses further supported the upregulated genes that were involved in ABA biosynthesis and signaling networks, and response to salt stress in iU1 plants. Collectively, these data indicated that GlcNAc1pUTs are essential for UDP-GlcNAc biosynthesis, protein N-glycosylation, fertility, and the response of plants to salt stress through ABA signaling pathways during seed germination and early seedling development.

## Introduction

Salt stress is one of the major abiotic stressors, which markedly inhibits plant growth and crop productivity. Salt stress, such as saline soil, has been one of the biggest threats to the security of food worldwide; in addition, food demand is increasing, but the arable land is decreasing. In plants, salt stress generates osmotic stress and ion cytotoxicity, which impair new shoot growth and enhance the senescence of mature leaves (Munns and Tester, 2008). Many major cellular processes, such as photosynthesis, protein and lipid synthesis, and energy metabolism, are affected by saline soil (van Zelm et al., 2020; Mansour and Hassan, 2022), and these effects further impair seed germination, plant growth and development. To cope with salt stress, plants must evolve elaborate systems to integrate complicated signaling networks (Deinlein et al., 2014; Zhu, 2016), and these systems perform tasks such as sensing cellular nutrient availability, activating plant stress hormone abscisic acid (ABA), and regulating protein modifications. These systems include pathways that involve changes in certain metabolic fluxes, which reflect the status of cells. One such pathway is the hexosamine biosynthetic pathway (HBP). HBP utilizes several energy units as its substrates, including glucose, acetyl-CoA, glutamine, and UTP, to synthesize its final product uridine diphosphate N-acetylglucosamine (UDP-GlcNAc), an active form of nucleotide sugar GlcNAc (Hanover et al., 2010; Chiaradonna et al., 2018). In this regard, HBP serves as an integrator of these major cellular metabolic pathways (carbohydrates, amino acids, fatty acids, and nucleotides) to better suit deleterious environments.

UDP-GlcNAc, a donor of GlcNAc, is synthesized through HBP, which is catalyzed by four successive reactions (Supplementary Figure S1); these processes are essential for cell growth and stress responses and are conserved across organisms (Milewski et al., 2006). The first committed step of HBP is the transamination of fructose-6-phosphate (Fru-6-P) and glutamine to form glucosamine-6-p (GlcN-6-P) and glutamate, and this step is catalyzed by a glutamine/fructose-6-phosphate amidotransferase (GFAT; Hassid et al., 1959; Durand et al., 2008). Subsequently, GlcN-6-P is acetylated from acetyl-CoA by a GlcN-6-P N-acetyltransferase (GNA) to generate N-acetylglucosamine-6-P (GlcNAc-6-P) and CoA (Nozaki et al., 2012). GlcNAc-6-P is then converted to GlcNAc-1-P by a phosphor-GlcNAc mutase. The final step is the GlcNAc-1-P + UTP ↔ UDP-GlcNAc + PPi reaction, which is catalyzed by an N-acetylglucosmine-1-P-uridylyltransferase (GlcNAc1pUT, Yang et al., 2010), or a UDP-N-acetylglucosamine pyrophosphorylase (UAP; Wang et al., 2015, 2021) to produce UDP-GlcNAc. GlcNAc1pUT and UAP are named after the forward and reverse catalytic reactions, respectively. Disruption of any enzyme activities of HBP virtually leads to severe cellular defects or even lethality in many organisms, such as yeast, mammals, and plants (Milewski et al., 2006; Chen et al., 2014; Pantaleon, 2015; Vu et al., 2019). In plants, defects in the HBP pathway may affect plant growth, alter abiotic stress sensitivity and induce defense responses (Nozaki et al., 2012; Wang et al., 2015, 2021).

Posttranslational modification is a fundamental cellular process that integrates internal and external stimuli and mediates dynamic protein functions. Protein N-glycosylation is among the most common modifications, which is essential for plant growth and stress responses and is conserved across eukaryotes (Banerjee et al., 2007; Bao and Howell, 2017; Nagashima et al., 2018). N-glycosylation begins with a supply of GlcNAc, which is the essential amino sugar moiety that initiates the processing of oligosaccharide precursor at the cytosolic side of the endoplasmic reticulum (ER; Nagashima et al., 2018). Initially, two GlcNAc molecules are transferred to a lipid-linked carrier, dolichylpyrophosphate (Dol-PP) by GlcNAc-1-phosphotransferase, and then, five mannose (Man) residues are sequentially added by mannosyltransferase to form Man_5_GlcNAc_2_-Dol-PP (Burda and Aebi, 1999). This oligosaccharide precursor is then transferred to the ER lumen for further modification (Pattison and Amtmann, 2009). Four more Man and three Glc residues are added to the precursor in an orderly manner to form the core oligosaccharide Glc_3_Man_9_GlcNAc_2_-Dol-PP, which is assembled by a series of membrane-bound glycosyltransferases (Snider et al., 1980; Helenius and Aebi, 2002; Nagashima et al., 2018). N-glycosylation occurs in the ER lumen through the transfer of core oligosaccharide to asparagine (Asn- or N-) in the Asn-X-Ser/Thr motif (X, any amino acid except Pro) of a nascent peptide, and this step is mediated by an oligosaccharyltransferase (OST) complex (Burda and Aebi, 1999; Pattison and Amtmann, 2009; Strasser, 2016). The N-linked core oligosaccharide is further processed through the sequential trimming of three Glc residues by glucosidases (Trombetta, 2003; Nagashima et al., 2018), and a Man residue is removed by the ER-α-mannosidase I (MNS3; Liebminger et al., 2009). The modified glycoprotein that is properly folded leaves the ER and moves into the Golgi apparatus, where it undergoes complex glycan modification before secretion (Strasser, 2016). N-glycan plays an important role in the folding, interaction, quality control, and secretion of proteins (Helenius and Aebi, 2001; Aebi, 2013; Shin et al., 2018). Defects in N-glycan processing often affect plant growth and stress responses or even cause lethality (Lane et al., 2001; Koiwa et al., 2003; Lerouxel et al., 2005; Zhang et al., 2009; Fanata et al., 2013; Bao and Howell, 2017; Nagashima et al., 2018).

In addition to N-glycosylation, O-linked glycosylation occurs in the Golgi apparatus and plays a critical role in the complex glycan processing (Strasser et al., 2014). The functions of O-linked glycoproteins include intracellular sorting or proteolytic activation and degradation (Seifert, 2020). O-GlcNAcylation is a single O-linked GlcNAc that is attached to the serine or threonine of the cytosolic and nuclear proteins, which are involved in many aspects of cellular processes, including transcription and translation regulation, signal transduction, metabolism, and development (Xu et al., 2017). When Arabidopsis SECRET AGENT (SEC), an O-GlcNAc transferase, loses functionality, multiple hormonal signaling pathways and plant growth and development, such as flowering, are affected through the impairment of O-GlcNAcylation of proteins, such as DELLA proteins, AtACINUS, AtPININ, and ARABIDOPSIS OF TRITHORAXI (ATX1), a histone lysine methyltransferase (HKMT; Hartweck et al., 2002; Zentella et al., 2016; Xing et al., 2018; Bi et al., 2021). Defects in O-GlcNAcylation homeostasis in mammals have been reported to be associated with diabetes, cancers, neurodegeneration, and even lethality (Marshall et al., 1991; McClain, 2002; Schimmelpfeng et al., 2006; Hanover et al., 2010; Slawson et al., 2010; Yang and Qian, 2017). Moreover, in addition to protein glycosylation, GlcNAc serves as a building block of glycoconjugates to modify sphingolipids (Ebert et al., 2018), glycosylphosphatidylinositol (GPI)-anchored proteins (Lalanne et al., 2004), a structural polymer of chitin in the yeast cell wall, an exoskeleton of arthropods or insect cuticles (Maia, 1994; Kato et al., 2002; Arakane et al., 2011).

To date, the functions of HBP-related genes have been largely characterized in plants. For instance, Arabidopsis *GFAT1* (At3g24090) is highly expressed in mature pollen but is undetectable in other tissues. No male gamete transmission is observed for the knockout *Atgfat1* mutant due to defects in the polar deposition of pectin and callose in the pollen cell wall. However, ROS accumulation, cell death, and protein underglycosylation were observed in the knockdown *Atgfat1* mutants grown on glucosamine (GlcN)-free media; these abnormal phenotypes can be rescued by exogenous GlcN. Moreover, transgenic plants overexpressing *AtGFAT1* exhibit enhanced GlcN production and resistance to tunicamycin (Tm), an ER stress-inducing agent (Vu et al., 2019). Mutation of Arabidopsis *GNA* (At5g15700), known as *lignescens* (*lig*), impairs plant growth and exhibits high-temperature sensitivity and ectopic lignin deposition under high-temperature (28°C) growth conditions. In addition, high temperature also causes a reduction in UDP-GlcNAc biosynthesis and protein N-glycosylation but induces the expression of *LUMINAL BINDING PROTEIN 3* (*BiP3*), a UPR marker gene, in the *lig* mutant compared to the wild-type plants (Nozaki et al., 2012). Similar to the *lig* mutant, the *GNA* mutation in rice, termed *Osgna1*, results in short roots and temperature sensitivity. The *Osgna1* mutant is deficient in UDP-GlcNAc and protein underglycosylation (Jiang et al., 2005); however, in contrast to *lig* that shows sensitivity to high temperatures, *Osgna1* exhibits more severe root growth defects at lower temperatures (25°C) than at higher temperatures (32°C). Two *GlcNA.UT*s, i.e., *GlcNA.UT1* (At1g31070) and *GlcNA.UT2* (At2g35020), which encodes GlcNAc1pUT1 and GlcNAc1pUT2, respectively, were cloned, and their catalytic activities were characterized in *Arabidopsis* (Yang et al., 2010). Both GlcNAc1pUT1 and GlcNAc1pUT2 utilize GlcNAc-1P, GalNAc-1P, and UTP as substrates to form UDP-GlcNAc, UDP-GalNAc and Pi. In addition, GlcNAc1pUT2 can also use Glc-1-P as a substrate to form its corresponding UDP-sugars (Yang et al., 2010). Mutation of *GlcNA.UT1* or *GlcNA.UT2* results in no conceivable phenotype; however, the double mutant is lethal. The double mutant with one normal *GlcNA.UT1* allele, i.e., *GlcNA.UT1*/*glcna.ut1 glcna.ut2*/*glcna.ut2*, which is derived from the F2 segregating population of the *glcna.ut1* x *glcna.ut2* cross, reveals the aberrant transmission of (*glcna.ut1* g*lcna.ut2*) gametes and defects in the male gametophytes at the pollen mitosis I stage; in addition, female gametophytes are arrested at the uninucleate embryo sac stage. Nevertheless, the double mutant with one normal *GlcNA.UT2* allele, i.e., *glcna.ut1*/*glcna.ut1 GlcNA.UT2*/*glcna.ut2*, arrests embryo development at the early globular stage (Chen et al., 2014). In rice, *GlcNA.UT* was named as UDP-N-acetylglucosamine pyrophosphorylase (*UAP*) or *SPOTTED LEAF 29* (*SPL29*), which was isolated from screening lesion mimic mutants (Wang et al., 2015). The *uap1*/*spl29* mutant displays early leaf senescence and defense responses in association with the accumulation of reactive oxygen species (ROS) and the induction of jasmonic acid and abscisic acid (ABA) biosynthesis (Wang et al., 2015, 2021). Furthermore, the *spl29-2* mutant plants accumulate uridine 5′-diphosphoglucose-glucose (UDPG) and induce the expression of ER stress- and UPR-related genes (Xiao et al., 2018). In contrast, the effect of *GlcNA.UT*s on UDP-GlcNAc biosynthesis in planta, protein N-glycosylation, and their response to abiotic stress remain less clear.

Because Arabidopsis *GlcNA.UT1* and *GlcNA.UT2* have functional redundancy and the double mutants are lethal, in this study, we thus used the RNA interference of *GlcNA.UT1* expression under the *glcna.ut2* knockout mutant background to generate transgenic plants, which are termed iU1s, and their biochemical and molecular functions were examined. The iU1 seedlings exhibited defects in UDP-GlcNAc biosynthesis and protein N-glycosylation under salt-stressed conditions. Moreover, iU1s were hypersensitive to salt and displayed characteristics including a delay in both seed germination and early seedling establishment. The salt sensitivity of iU1s was associated with the induction of ABA biosynthesis and signaling networks. The salt-sensitive phenotypes in iU1s can be abrogated by exogenous treatment with the ABA biosynthesis inhibitor fluridone or through introducing an ABA-deficient mutant allele *nced3* (Wan and Li, 2006) into iU1s. These data provide evidence that Arabidopsis GlcNAc1pUTs are essential for UDP-GlcNAc production, protein N-glycosylation and ABA-mediated salt stress response during germination and early seedling growth.

## Materials and Methods

### Plant Materials and Growth Conditions

*Arabidopsis thaliana* ecotype Columbia (Col-0) was used in this study, and information on the mutant lines used in this study is described in Supplementary Table S1. Unless described otherwise, all seeds were sterilized and subjected to a cold pretreatment at 4°C for 3 days in the dark before being sown on soil or MS plates, which contain half strength of MS basal salts, B5 organic compounds, 1% sucrose, and 0.05% MES [2-(N-morpholino)ethanesulfonic acid monohydrate]. After adjusting the pH to 5.7, 7 g/l phytoagar (Duchefa Biochemie) was added. The plants grown on agar plates were under a 16/8 h day/night photoperiod with a light intensity of ~50 μmol m^−2^ s^−1^. The plants grown in soil were under the same photoperiod with a light intensity of ~80–100 μmol m^−2^ s^−1^. The germinated seeds were defined as the radical roots with erupted testa at least 0.5 mm in length. In this study, at least three biological replicates were performed in each experiment. Unless stated otherwise, the quantified data represent the means ± SD of at least three biological replicates.

### Immunoblot Analyses

The polyclonal antibodies were raised in rabbits against the synthetic peptides of AtPDI5 (amino acids 476–491: FVDKNKDTVGEPKKEE; Andème Ondzighi et al., 2008) and GlcNAc1pUT1 (amino acids 104–121: TVDGRTMEDREKWWKMGL). Affinity-purified polyclonal antibodies were tested by immunoblot analyses. Other antibodies or lectin were purchased from commercial companies as follows: anti-HRP (Sigma-Aldrich), anti-GlcNAc1pUT2 (known as anti-UAGPase in AGRISERA), and Concanavalin A (EY Lab). Proteins were extracted by homogenizing tissues or seedlings with Protein Extraction Buffer (50 mM Tris–HCl pH 7.5, 300 mM NaCl; 5 mM EDTA pH 8.0, 1 mM DTT, 1% Triton X-100, Roche cOmplete EDTA-free Protease Inhibitor Cocktail). Samples were centrifuged at 13,000 × g, and 4°C. After the protein concentration was determined by a Pierce BCA protein assay kit (Thermo Fisher), proteins were denatured by NuPAGE LDS sample buffer (Thermo Fisher). Protein samples were separated by NuPAGE Bis-Tris SDS protein gel (Thermo Fisher) and then transferred to polyvinylidene fluoride (PVDF) membranes, followed by stepwise incubations with primary antibodies, secondary antibodies and chemiluminescent detection (SuperSignal West Pico PLUS, Thermo Fisher) according to the manufacturer’s instructions.

### Nucleotide Sugar Analysis

The extraction of nucleotide sugar was performed based on the method of Nozaki et al. (2012) with modifications. In brief, 0.1 g of frozen seedlings was homogenized and extracted with 0.8 ml of extraction buffer (75% ethanol, 25% of 150 mM NaCl in 10 mM sodium-phosphate buffer pH 7.4) containing 2 ng cytidine-5′-monophospho-N-acetylneuraminic acid (CMP-NeuAc) sodium salt (Sigma, C8271) as the internal control. After centrifugation at 16,000 × g at 4°C for 10 min, the supernatant was transferred to a new tube and lyophilized. The lyophilized sample was redissolved in 1 ml of H_2_O. The Envi-Carb SPE column (Supelco, 57,109-U) was conditioned by 1 ml of elution buffer (60% acetonitrile containing 0.3% formic acid, adjusted pH to 9.0 with ammonia) and then, equilibrated with 1 ml of H_2_O. The 1 ml sample solution was loaded onto the column and the flow-through was discarded. After washing the column with 1 ml of H_2_O, the column was eluted with 1 ml of elution buffer. Following lyophilization of the eluent, the sample was reconstituted with 100 μl of 50% acetonitrile. An ACQUITY UltraPerformance LC^®^ (UPLC^®^) system (Waters, Milford, MA, United States) coupled to an Orbitrap Elite (Thermo Scientific) mass spectrometer (MS) was used for analysis by a HESI interface. The chromatographic separation for samples was carried out on an ACQUITY UPLC BEH Amide Column, 1.7 μm, 2.1 mm × 100 mm column (Waters). The column was maintained at a temperature of 25°C, and 5 μl of the sample was injected per run. Mobile phase A was 40% v/v acetonitrile with 20 mM ammonium acetate pH 9.0, and mobile phase B was 95% v/v acetonitrile with 20 mM ammonium acetate pH 9.0. Gradient elution with a flow rate of 0.4 ml/min was performed with a total analysis time of 6 min. The gradient included 80% B at 0 min, 0.1% B at 3 min, a hold at 0.1% B until 3.5 min, 80% B at 3.51 min, and a hold at 80% B until 6 min. The general instrumental conditions included sheath gas, auxiliary gas, and sweep gas of 30, 15, and 2 arbitrary units, respectively; an ion transfer tube temperature of 360°C; a vaporizer temperature of 350°C; and a spray voltage of 2,500 V for negative mode. For analysis, a full MS scan mode with a scan range m/z 150–615, and a resolution of 15,000 was applied. Xcalibur 4.1 software (Thermo Scientific) was used for data processing.

### ABA Assay

For ABA extraction, frozen samples were homogenized, and 0.5 ml extraction solvent (2-propanol/H_2_O/concentrated HCl = 2:1:0.002) containing 100 ng isotopically labeled d6-ABA was added as an internal standard and incubated at 4°C for 30 min with gentle agitation. Then, 1 ml of dichloromethane was added and agitated for another 30 min at 4°C, followed by centrifugation at 13,000 x g for 5 min at 4°C. The bottom phase was transferred to a new tube and desiccated by a SpeedVac. For the seedling samples, the samples were then dissolved in 60 μl of 80% methanol and subjected to LC/MS analysis. For the germinating seed samples, the desiccated samples were dissolved in 1 ml 5% ammonia solution. The Oasis MAX column (Waters, SKU186000367) was conditioned by 1 ml methanol twice and then equilibrated by 1 ml H_2_O twice, followed by 1 ml 5% ammonia twice. The sample solution dissolved in 5% ammonia was loaded onto the column, followed by the subsequent washing steps: 1 ml 5% ammonia twice, 1 ml H_2_O twice, and 1 ml methanol twice. Finally, the column was eluted twice with 1 ml methanol containing 10% formic acid. The eluent was then desiccated by a SpeedVac and dissolved in 60 μl 80% methanol for LC/MS analysis. The LC system used for analysis was an ultra-performance liquid chromatography (UPLC) system (ACQUITY UPLC, Waters, Milford, MA). The sample was separated with an ACQUITY UPLC HSS T3 column (1.8 μm particle size, 2.1 mm × 100 mm, Waters). The flow rate was 0.3 ml/min with an injection volume of 10 μl and a column temperature of 30°C. The composition of mobile phase A was water containing 0.1% acetic acid, and phase B was methanol containing 0.1% acetic acid. The UPLC system was coupled online to a Waters Xevo TQ-XS triple quadrupole mass spectrometer (Waters, Milford, United States). D6-ABA was used as an internal standard. Characteristic MS transitions were monitored using negative multiple reaction monitoring (MRM) mode for ABA (m/z 263 > 153) and D6-ABA (m/z 269 > 159). Data acquisition and processing were performed using MassLynx version 4.2 and TargetLynx software (Waters Corp.).

### Microarray Analysis

Total RNA was extracted from the seedlings of Col-0 and iU1-52 that were grown in MS medium with or without 200 mM NaCl for 14 days. After the RNA was reverse-transcribed and labeled, the products were pooled and hybridized to an Agilent Arabidopsis V4 4*44 K Microarray (Agilent Technologies, United States). The microarrays were scanned with an Agilent microarray scanner at 535 nm for Cy3. The array image was analyzed by the Feature Extraction software version 10.7.1.1 using the default setting. The resulting CEL files were analyzed using GeneSpring (version 14.3, Agilent Single Color Technology). The raw signal values employed a cutoff if the values in all samples were <100. Following the cutoff, quantile normalization and baseline transformation to the median of all samples were performed. The normalized genes were then statistically analyzed using two-way ANOVA, and the *p* values were computed with multiple testing corrections of the Benjamini–Hochberg False Discovery Rate (value of *p* of 0.05). Three biological replicates were performed in this experiment. The raw data are available in the Gene Expression Omnibus (GEO) database under Accession No. GSE193841. Gene expression that passed 2-way ANOVA (*p* < 0.05) was applied to fold change (FC) comparisons.

### RT-qPCR and Genomic DNA PCR

Total RNA was extracted from 14-day-old seedlings that were grown on half-strength MS plates supplemented with or without 200 mM NaCl. The extraction and RT-qPCR were performed as described previously (Chen et al., 2014). The primers used for RT-qPCR and genomic DNA PCR are listed in Supplementary Tables S2, S3, respectively.

## Results

### RNA Interference Impairs the Expression of *GlcNA.UT1* and Causes Sterility in iU1 Lines

**Figure 1:**
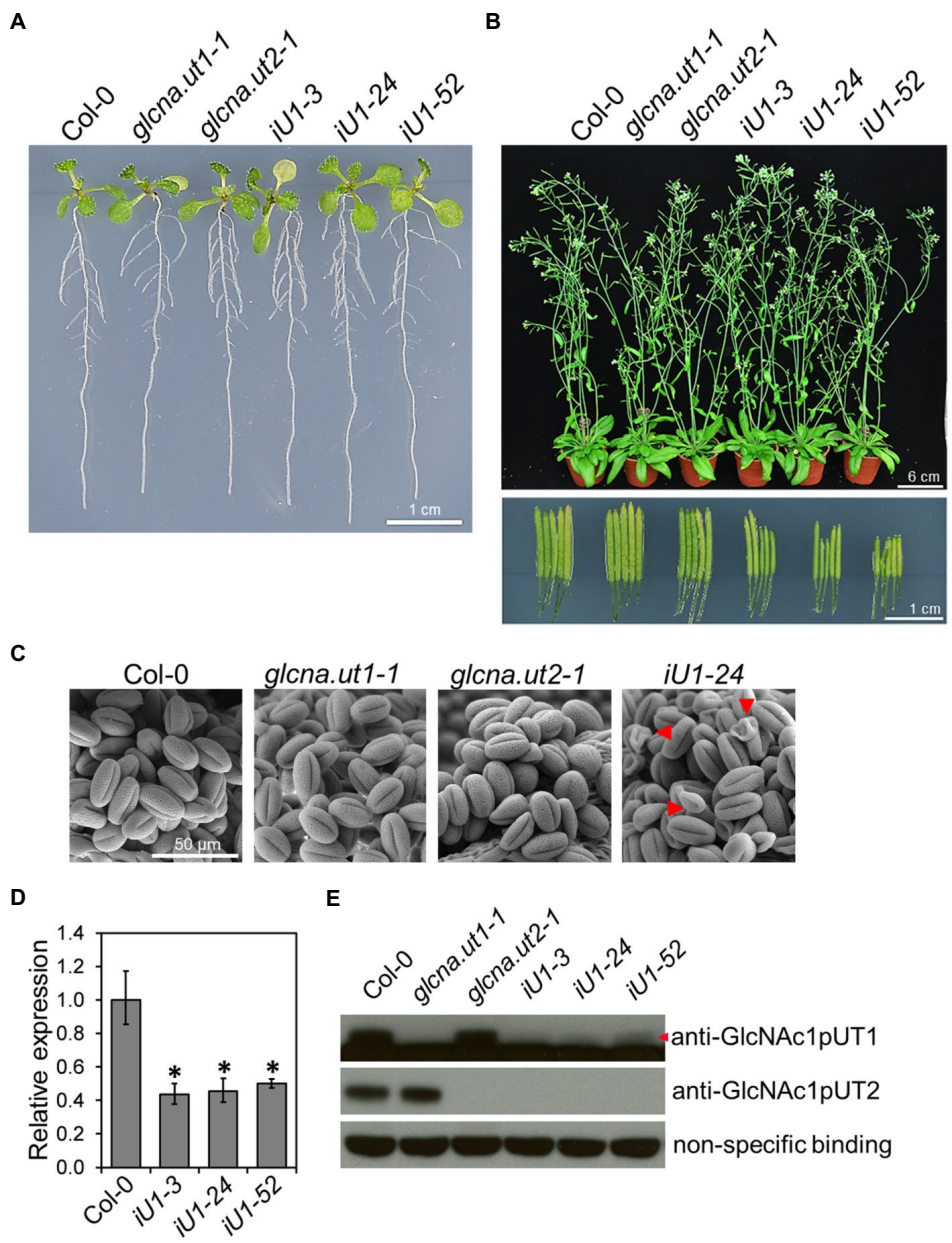
Phenotypic and molecular analyses of *glcna.ut* single mutants and iU1 transgenic plants. **(A)** Seedling phenotype comparison. Seedlings were grown on MS medium for 10 days. iU1 lines represent dsRNAi interference of *GlcNA.UT1* in the *glcna.ut2-1* mutant background. **(B)** Comparison of the plant phenotype and silique length. Plants were grown in soil for 37 days. **(C)** Pollen morphology. Pollen grains were derived from the plants shown in **(B)** and visualized by scanning electron microscopy. Shrunken pollens are indicated by red arrowheads. **(D,E)** Expression of *GlcNA.UT1.* Seedlings grown in MS medium for 14 days were used for total RNA or protein extraction, followed by RT-qPCR **(D)** and Western blot **(E)** analyses. The values in **(D)** represent the means ± SD of four biological replicates, each with technical triplicates. ^*^p < 0.01. Student’s *t*-test. For Western blot analysis, equal amounts of proteins were loaded in each lane and a nonspecific binding band was used as a loading control. GlcNAc1pUT1 proteins are indicated by a red arrowhead.

The *Arabidopsis* genome contains two *GlcNA.UT* genes, which are named *GlcNA.UT1* and *GlcNA.UT2* (Yang et al., 2010). Mutation of *GlcNA.UT1* or *GlcNA.UT2* gene displays no conceivable phenotype; however, the double mutants are lethal, suggesting that the functional redundancy of these genes is essential for plant growth (Chen et al., 2014). To further investigate the function of *GlcNA.UT* genes in UDP-GlcNAc biosynthesis, protein N-glycosylation, and abiotic stress response, we used the double-stranded RNA interference (dsRNAi) approach to knock down the expression of *GlcNA.UT1* in the *glcna.ut2* null mutant backgrounds. The dsRNAi *GlcNA.UT1*/*glcna.ut2* transgenic seedlings, termed iU1, that were grown on agar plates for 10 days displayed a seedling phenotype similar to that of the wild type (Figure 1A). Although the mature plants in iU1s that were grown in soil for 37 days showed no conceivable phenotype (Figure 1B, upper panel), they displayed short siliques (Figure 1B, lower panel) and abnormal pollen grains (Figure 1C) compared to that of the wild type and the single mutants, *glcna.ut1-1* and *glucan.ut2-1*. The iU1 plants reduced the expression of *GlcNA.UT1* transcript and protein under the *glcna.ut2* background (Figures 1D,E), indicating that the targeted gene expression was effectively knocked down by dsRNAi. We observed that the iU1 phenotype is similar to the heterozygous double mutant *GlcNA.UT1*/*glcna.ut1 glcna.ut2*/*glcna.ut2* and displayed fertility defects (Chen et al., 2014). As the heterozygous double mutants *GlcNA.UT1*/*glcna.ut1 glcna.ut2*/*glcna.ut2* were only identified by genotyping the F2 segregating population that was derived from crosses of *glcna.ut1* x *glcna.ut2*, it was difficult to use them for further experiments because segregation occurred in the offspring of these heterozygous double mutants. Thus, the iU1 transgenic plants were used in this study because their offspring phenotypes are stable.

### The iU1 Transgenic Plants Confer Hypersensitivity to Salt Stress

**Figure 2:**
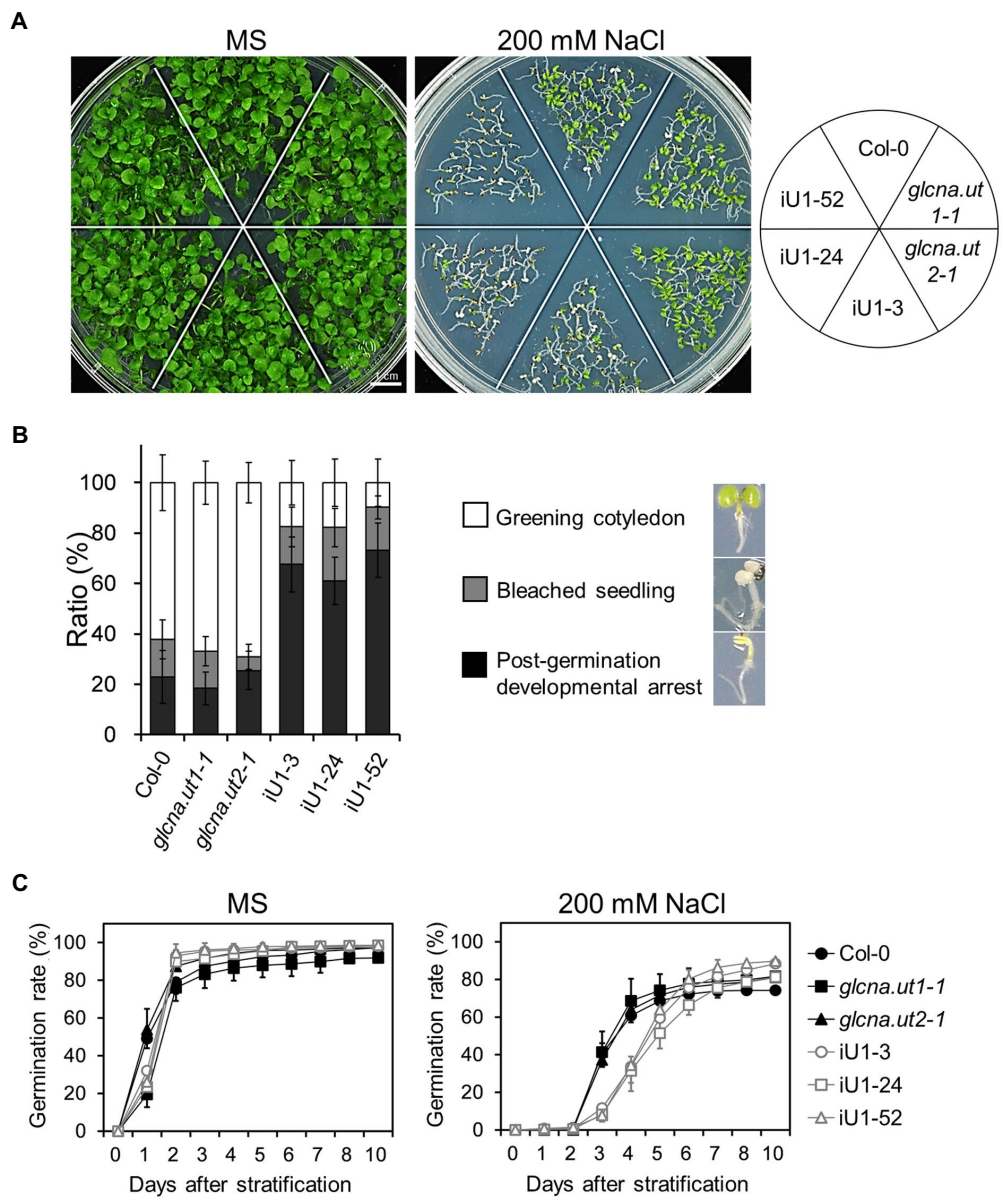
Salt hypersensitivity of the iU1 transgenic plants. **(A)** Salt hypersensitivity. Seedlings were grown on MS or 200 mM NaCl-containing media for 14 days. **(B)** Analyses of the salt-treated seedling phenotypes. Data are presented as the means ± SD of at least eight biological replicates. Each replicate contained ~45 seeds. **(C)** Seed germination. Data are presented as the means ± SD of at least 12 biological replicates. Each replicate contained ~40 seeds.

The hexosamine biosynthetic pathway provides nucleotide sugars that are indispensable for organisms, including bacteria, humans and plants. The role of HBP in stress response in mammalian cells has been considerably discussed (Horn et al., 2020), yet it is less clear in plants. To examine whether HBP is involved in the abiotic stress response, iU1 seeds were grown on agar plates supplemented with salt stress. Under normal growth conditions, seed germination and the seedling phenotype in iU1s were generally similar to those of the wild type and single mutants (Figures 2A,C, left panels). Under 200 mM NaCl, the iU1 seedlings showed hypersensitivity to salt as seed germination was delayed (Figure 2C, right panel) and the cotyledons were unexpanded and nongreening, i.e., postgermination developmental arrest (Figures 2A,B). In addition to seed germination delay, the iU1 seeds also displayed a slightly higher germination rate than that in wild type. The mechanism underlying the iU1 seeds showing a higher germination rate than the wild-type seeds remains unknown. One possibility is that the iU1 lines contain fewer inactive seeds relative to wild type so that they display a higher germination rate. However, under salt stress conditions, the iU1 root elongation was similar to that of the wild-type and single mutant plants except that *stt3a* (*stt3a-2*), a mutation of a catalytic subunit of the oligosaccharyltransferase (OST) complex, showed a shorter root length than that in the wild type at 140 and 160 mM NaCl concentrations (Koiwa et al., 2003; Supplementary Figure S2), but the seed germination rate and early seedling establishment were similar between the wild type and *stt3a-2* (Supplementary Figures S2C–E). However, the salt-sensitive phenotypes of iU1 seedlings were enhanced along with the increased salt concentrations in the media (Supplementary Figure S3A), particularly at 200 mM NaCl, a concentration that most iU1 seedlings displayed postgermination developmental arrest.

Considering the possibility of off-target effects of the dsRNAi strategy, we verified whether the salt-hypersensitive phenotypes of iU1 lines primarily resulted from the knockdown of *GlcNA.UT1* rather than that of other genes. To test this hypothesis, we introduced functional *GlcNA.UT2* alleles into the iU1 line by crossing the iU1-52 with the *glcna.ut1-1* single mutant, in which the *GlcNA.UT2* allele is normal (Chen et al., 2014). Given that *GlcNA.UT1* and *GlcNA.UT2* are functionally redundant, the introduction of the *GlcNA.UT2* allele is sufficient to repress the salt hypersensitivity caused by the iU1. As shown in Supplementary Figures S3B,C, the salt-stressed phenotypes were relieved in the iU1-52 x *glcna.ut1-1* seedlings, indicating that *GlcNA.UT2* is involved in salt hypersensitivity but not other genes. However, considering that the knockout mutant *glcna.ut2* showed no phenotype (Figure 2; Chen et al., 2014) and that the combination of *glcna.ut2* with dsRNAi *GlcNA.UT1* in iU1s displayed salt hypersensitivity, these data indicate that the salt-sensitive phenotypes observed in iU1s are attributable to functional defects in both *GlcNA.UT1* and *GlcNA.UT2*.

To further examine whether the salt hypersensitivity observed in iU1s is due to osmotic stress response, we sowed seeds on media supplemented with 400 mM mannitol, which provides a similar osmotic strength as that of 200 mM NaCl. The results indicated that the iU1 seeds also showed a lower seed germination rate and less seedling establishment than those in the wild-type seedlings (Supplementary Figures S4A,C). However, the ratio of postgermination developmental arrest was lower in 400 mM mannitol than in 200 mM NaCl in iU1 seedlings (Supplementary Figure S4B vs. Figure 2B). These data indicate that salt hypersensitivity in iU1s is partially due to osmotic stress effects.

### Defects in GlcNAc1pUTs Reduce UDP-GlcNAc Biosynthesis Under Salt Stress

**Figure 3:**
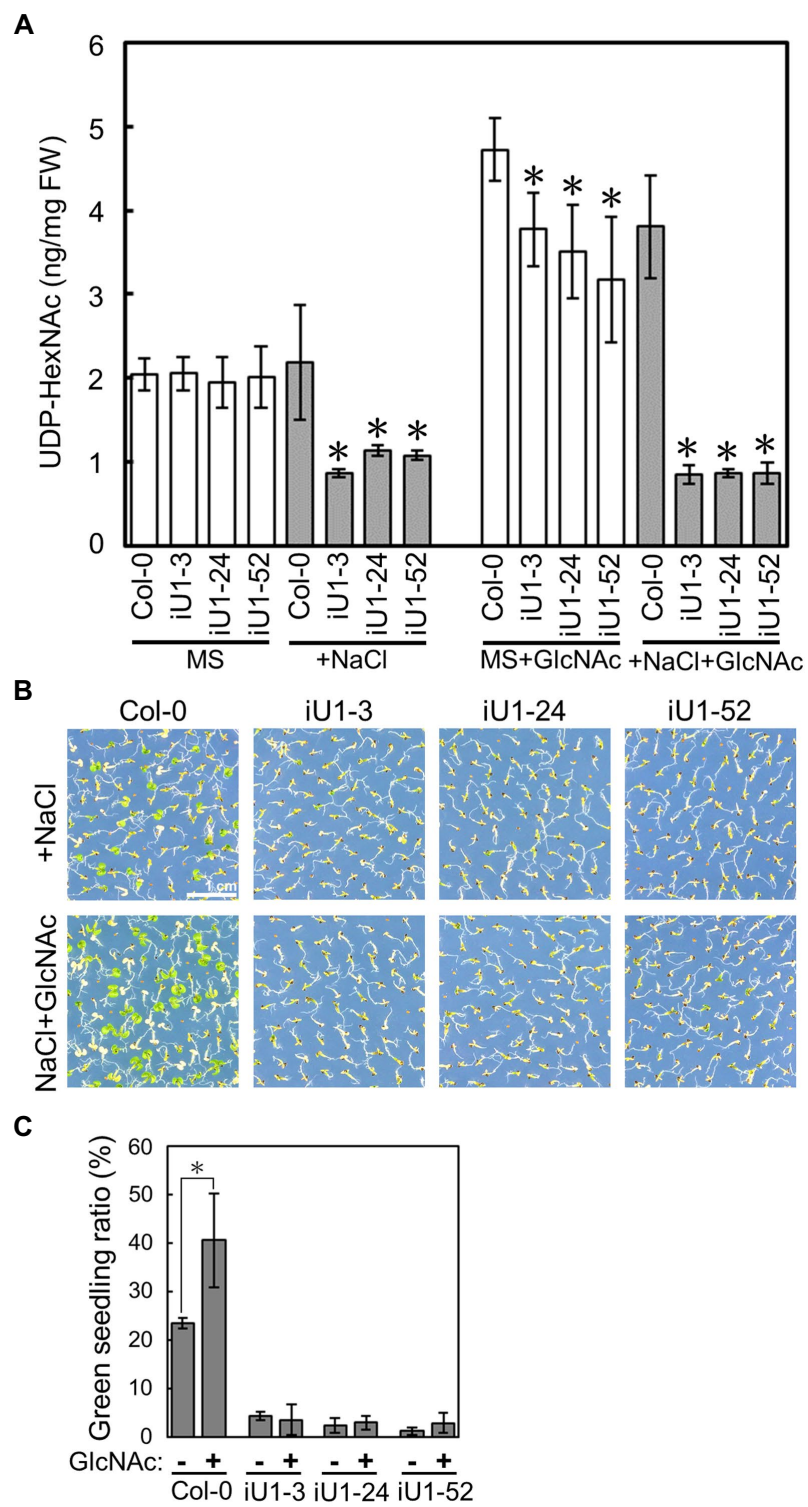
Reduced contents of UDP-N-acetylhexosamines (UDP-HexNAc) in salt-treated iU1 seedlings. **(A)** Reduction in UDP-HexNAc in iU1 seedlings. UDP-HexNAc was analyzed by LC/MS from 14-day-old seedlings grown on MS agar media supplemented with or without NaCl (200 mM) or NaCl + GlcNAc (100 μM). Data represent the means ± SD of four biological replicates. ^*^p < 0.05. Student’s *t*-test. **(B)** Phenotypic comparison. Seedlings were grown under the same conditions as those shown in **(A)**. **(C)** Seedlings with greening cotyledons in **(B)** were quantified. Data represent the means ± SD of at least four biological replicates, each with 60–80 seeds. ^*^p < 0.05, Student’s *t*-test.

*GlcNA.UT*s encode the protein GlcNAc1pUTs that catalyze the last step of HBP to produce UDP-GlcNAc (Yang et al., 2010). As shown in Figure 1, the expression of both *GlcNA.UT1* transcript and GlcNAc1pUT1 protein was reduced, and GlcNAc1pUT2 protein was undetectable in iU1 transgenic plants. We further examined the effect of dsRNAi *GlcNA.UT1*/*glcna.ut2* in iU1 plants on the UDP-GlcNAc biosynthesis under normal and salt stress conditions. Previous studies have demonstrated that HBP can synthesize UDP-GlcNAc and UDP-GalNAc (Yang et al., 2010; Nozaki et al., 2012). For example, GlcNAc1pUTs can utilize GlcNAc-1-P and N-acetylgalactosamine (GalNAc-1-P) as substrates, and GNA can utilize N-acetylglucosamine (GlcNAc) and N-acetylgalactosamine (GalNAc) to synthesize UDP-GlcNAc and UDP-GalNAc, respectively (Yang et al., 2010; Nozaki et al., 2012). In addition, relative to UDP-GalNAc, UDP-GlcNAc is the primary form of UDP-N-acetylhexosamine in *Arabidopsis* (Nozaki et al., 2012). As UDP-GlcNAc and UDP-GalNAc cannot be distinguished by our liquid chromatography (LC) coupled with mass spectrometry (MS), we thus used LC/MS to assay both UDP-GlcNAc and UDP-GalNAc, which is termed UDP-N-acetylhexosamine (UDP-HexNAc). Fourteen-day-old seedlings that were grown on agar plates were used to assay the levels of UDP-HexNAc. The data indicated that similar UDP-HexNAc contents were observed between the wild-type seedlings and three independent lines of iU1 seedlings when grown under normal conditions; however, UDP-HexNAc was significantly reduced in iU1s but not in the wild-type seedlings under salt stress (200 mM NaCl; Figure 3A). Moreover, the exogenous application of N-acetylglucosamine (GlcNAc), a substrate of N-acetylglucosamine kinase (GNK) that catalyzes GlcNAc to form GlcNAc-6P in the HBP salvage pathway (Supplementary Figure S1; Furo et al., 2015), increased the biosynthesis of UDP-HexNAc in both the wild-type and iU1 seedlings under normal growth conditions, i.e., MS + GlcNAc; however, under NaCl + GlcNAc, UDP-HexNAc biosynthesis was inhibited in the iU1s compared to the wild-type plants (Figure 3A). In addition, the exogenous application of GlcNAc improved the growth of wild-type seedlings but not iU1s under salt stress conditions (Figures 3B,C). These data indicate that UDP-HexNAc biosynthesis in iU1s is limited under salt stress due to defects in the GlcNAc1pUT1 and GlcNAc1pUT2 proteins.

### Salt Stress Alters N-Linked Glycosylation and Induces ER Stress in iU1 Plants

**Figure 4:**
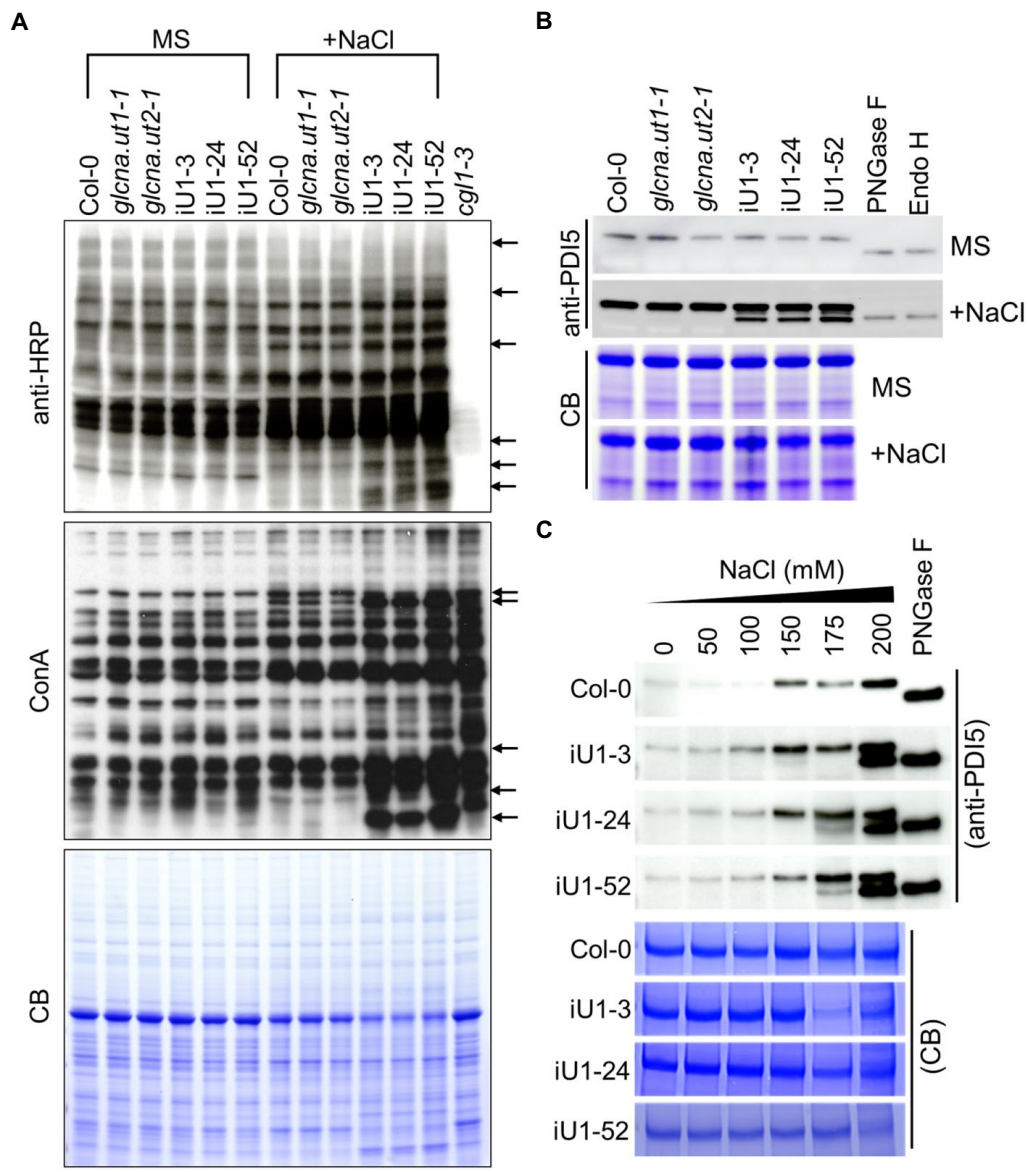
Protein N-glycosylation is altered in iU1 seedlings under salt stress. **(A)** Immunoblot analysis of N-glycoprotein profiles. The blots were hybridized with anti-HRP antibody or ConA (Concanavalin A, a jack bean lectin). Arrows indicate variations in band intensity between Col-0 and the iU1 lines under salt treatment. The *cgl1-3* mutant proteins were loaded as an N-glycosylation control. **(B,C)** Immunoblot analyses reveal the change in the glycosylated state of PDI5 under 200 mM NaCl **(B)** or other salt concentration treatments **(C)**. PNGase F and Endo H are enzymes that can remove N-glycan chains from glycoproteins and produce unglycosylated proteins. Total proteins were extracted from 14-day-old seedlings subjected to NaCl treatment. At least three independent experiments were performed in this study, and consistent results were obtained. Coomassie blue (CB) staining was used as a loading control.

UDP-GlcNAc is the donor of GlcNAc that is a sugar moiety involved in the initiation of N-glycan processing for protein glycosylation. Thus, a deficit of UDP-GlcNAc might interrupt N-glycan formation and protein N-glycosylation. To test this hypothesis, concavannaA (ConA), which is a lectin that recognizes the terminal high-mannose type of glycans (von Schaewen et al., 1993), and horseradish peroxidase antibody (anti-HRP), which can recognize complex-type N-glycans (Faye et al., 1993), was applied to detect the N-linked glycoproteins. The results indicated that similar glycoprotein patterns were detected either by ConA or anti-HRP among the genotypes tested under normal growth conditions (i.e., MS medium); in contrast, some differences in banding patterns between the wild type, single mutants, and iU1s were observed in ConA or anti-HRP assays under salt stress (200 mM NaCl) conditions (Figure 4A, arrows). The *complex glycan less 1–3* (*cgl1-3*) mutant that shows a severe defect in complex N-glycans (von Schaewen et al., 1993) was used as an internal control. To further confirm the specific defect in N-glycoproteins in iU1 plants, a typical N-linked glycoprotein, protein disulfide isomerase 5 (PDI5), which is commonly used as an N-glycosylation indicator, was examined under both normal growth and salt-stressed conditions. The Western blots indicated that the PDI5 protein showed a single band, which represented the glycosylated form. After N-glycans were removed by peptide/N-glycosidase F (PNGaseF) or endoglycosidase H (Endo H; Tarentino et al., 1974; Tretter et al., 1991), the unglycosylated PDI5 protein showed a band shift with a lower molecular mass. In contrast, the induction of unglycosylated PDI5 proteins in iU1s was observed in the salt-treated seedlings but not in the wild-type and single mutants (Figure 4B). The unglycosylated PDI5 proteins appeared under salt stress, with concentrations ranging from 150 to 200 mM NaCl, and the band intensity was more pronounced at 200 mM NaCl in iU1s (Figure 4C). Likewise, two membrane-associated myrosinases, β-thioglucoside glucohydrolase 1 (TGG1) and TGG2, largely contained similar protein levels among the genotypes tested under normal growth conditions; however, under salt stress, the expression of glycosylated TGGs was suppressed; particularly in iU1, and the unglycosylated TGG proteins were increased in iU1s (Supplementary Figure S5, arrowheads). These data demonstrated that the reduction in UDP-GlcNAc production in iU1s may cause protein underglycosylation under salt-stressed conditions.

**Figure 5:**
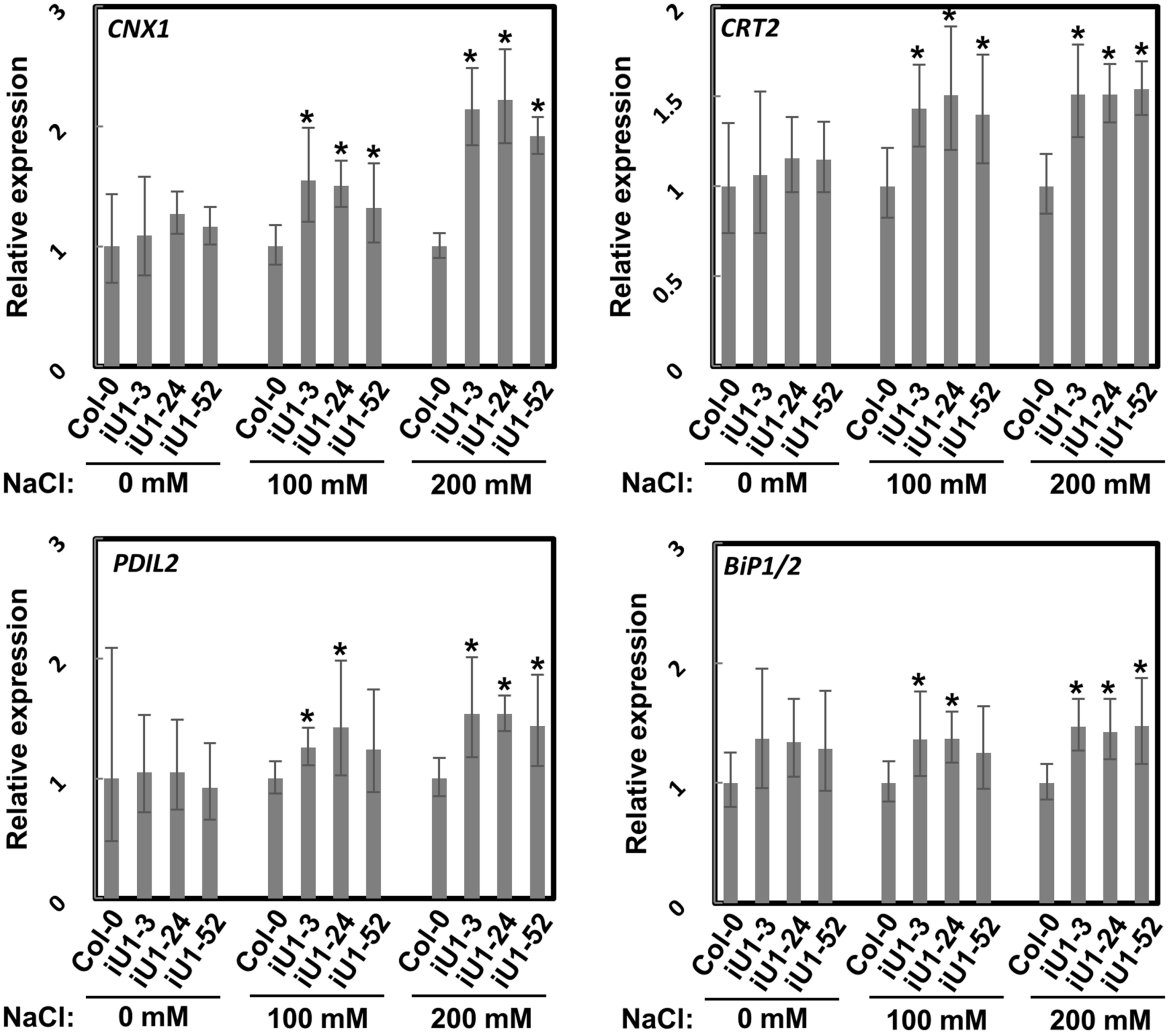
Upregulation of ER stress marker genes in iU1s under salt stress. Seedlings grown on MS agar plates supplemented with or without salt stress for 14 days were used for total RNA extraction, followed by RT-qPCR. Data are normalized to *PP2A* and presented as the means ± SD of four biological replicates, each with technical triplicates. ^*^p < 0.05, Student’s *t*-test.

N-glycosylation is an important posttranslational modification essential for protein folding in the endoplasmic reticulum (ER). Proteins in the ER that are incorrectly folded or unfolded due to stress will not be exported to their destinations. The accumulation of these improperly folded proteins causes ER stress and induces the unfolded protein response (UPR), which further triggers specific gene expressions for the release of ER stress. To test whether ER stress occurs in iU1s under salt stress, the expressions of several ER stress marker genes, *calnexin 1* (*CNX1*), *calreticulin 2* (*CRT2*), *PDI-like 2* (*PDIL2*), and heat shock *binding proteins 1* and *2* (*BiP1*/*2*), were evaluated. Arabidopsis *BiP1* and *BiP2* have nearly identical nucleotide sequences; thus, the primers used could not distinguish them (Liu and Howell, 2010). Reverse transcription-quantitative polymerase chain reaction (RT-qPCR) analysis showed that the expression of these genes was largely upregulated in the seedlings of iU1s compared to the wild type under salt stress conditions (Figure 5). These data indicated that N-linked glycosylation of proteins is impeded in the salt-treated iU1 seedlings, and this process is associated with the induction of the ER stress.

### Tunicamycin Enhances the Inhibition of Seedling Growth Under Salt Stress

Defects in the early steps of protein N-glycosylation may cause salt-inhibited root elongation (Koiwa et al., 2003; Kang et al., 2008). To further examine whether salt hypersensitivity in iU1s is the consequence of defects in protein N-glycosylation, tunicamycin (Tm), an ER stress inducer agent that blocks the transfer of GlcNAc to the ER membrane-associated lipid dolichol and inhibits N-glycan processing and protein N-glycosylation (Hori and Elbein, 1981), was used in this study. The treatment with Tm indeed reduced protein N-glycosylation, as detected by ConA (Supplementary Figure S6A). However, Tm did not affect the seed germination rate in general (Supplementary Figure S6B) in the wild-type and iU1-52 seedlings under normal and salt-stressed growth conditions. Moreover, the Tm treatment at 100 ng/ml inhibited seedling growth among the genotypes tested, but most seedlings were bleached at the 200 ng/ml concentration in the absence of salt stress (Supplementary Figure S6C) after 14 days of culture. However, the Tm treatments enhanced the bleached seedlings of the wild-type, single mutants and iU1s in the presence of salt stress (Supplementary Figures S6C,D). Thus, the Tm treatment enhances the salt inhibition of seedling growth.

### iU1 Seedlings Show ABA Sensitivity and Accumulation Under Salt Stress

**Figure 6:**
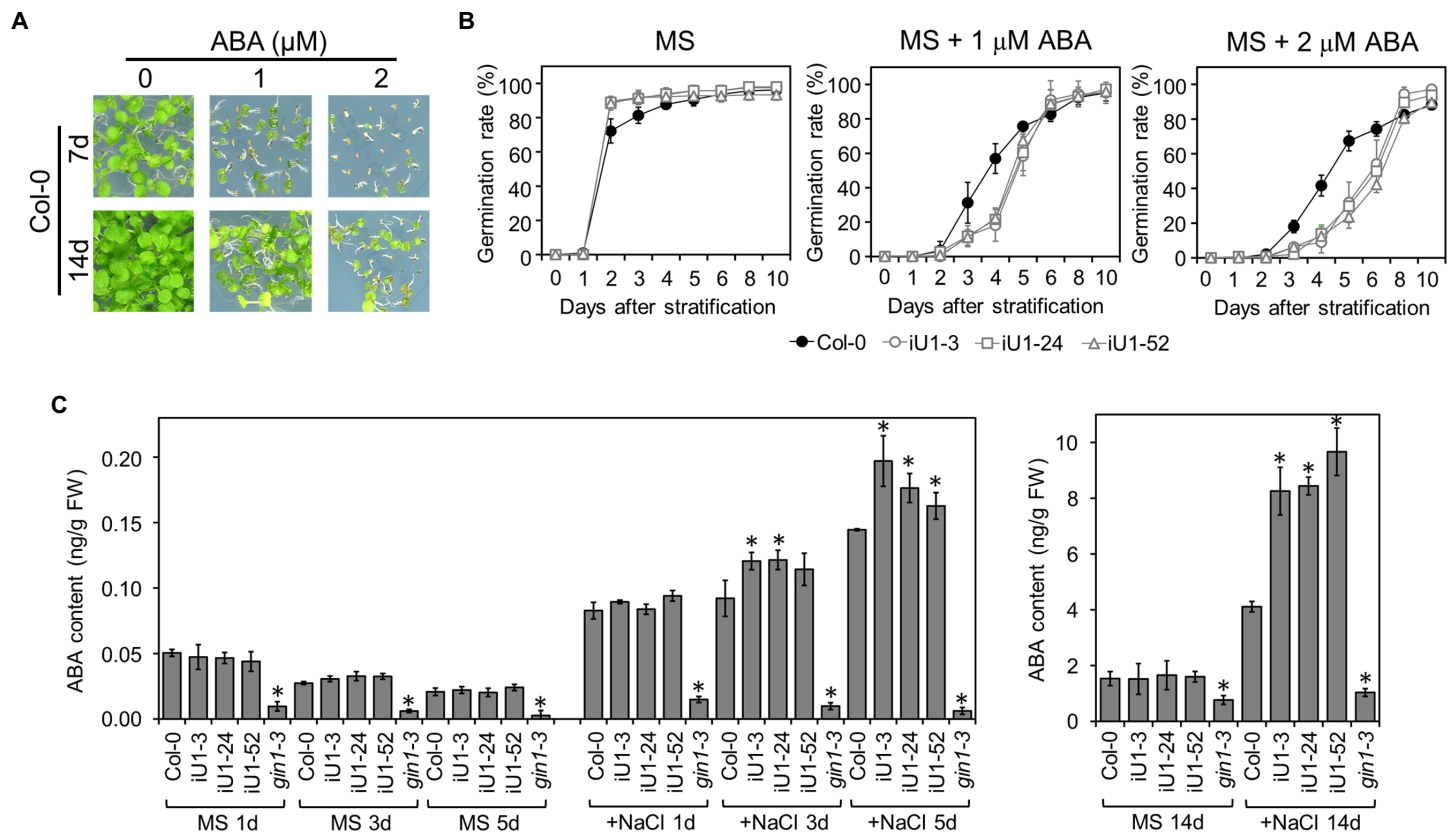
The iU1 plants increased the ABA contents under salt treatments. **(A)** Inhibition of seed germination by exogenous ABA. **(B)** Seed germination rate. Seeds were sown on agar plates supplemented with or without ABA. Data are presented as the means ± SD of at least 12 biological replicates. Each replicate contained ~40 seeds. **(C)** ABA contents of germinating seeds. Seeds germinated on MS or 200 mM NaCl medium for 1, 3, 5, or 14 days were harvested, and ABA was extracted and analyzed by LC/MS. The ABA-deficient mutant *gin1-3* (also known as *aba2*) was used as an internal control. Data represent the means ± SD of three or four biological replicates. ^*^p < 0.05, Student’s *t*-test.

Abscisic acid (ABA) is the major plant stress hormone, and its biosynthesis is significantly induced under salt and drought stresses. ABA inhibits seed germination and arrests seedling development (Figure 6A). Moreover, iU1 seed germination was more sensitive to ABA than that of wild type (Figure 6B), and this was similar to the salt stress results (Figure 2C). To examine whether the salt hypersensitivity observed in iU1s is correlated with the function of ABA, the ABA assay was employed in the seedlings of wild type and iU1s grown under normal or salt stress conditions. The results indicated that the ABA levels showed a slight reduction over the days of germination, and there was no significant difference between Col-0 and iU1s when the seedlings grew on MS agar plates for one to 5 days; however, under salt stress for 3 or 5 days, the ABA levels were higher in iU1s than in the wild-type seedlings (Figure 6C, left panel). After 14 days of culture, the ABA levels remained similar between the wild-type and iU1 seedlings under normal growth conditions; in contrast, the ABA levels increased in both the wild-type and iU1 seedlings and were significantly higher in iU1s than in the wild-type seedlings under salt-stressed conditions (Figure 6C, right panel). In this assay, the ABA-deficient mutant *glucose insensitive 1–3* (*gin1-3*), an *aba2* mutant allele (Cheng et al., 2002), was used as an internal control and exhibited low levels of ABA contents under normal or salt stress conditions. These data indicated that the salt hypersensitivity of iU1s is associated with higher levels of ABA.

### Exogenous Fluridone and the Introduction of a *nced3* Mutant Allele Abrogate Salt Sensitivity in iU1 Seedlings

**Figure 7:**
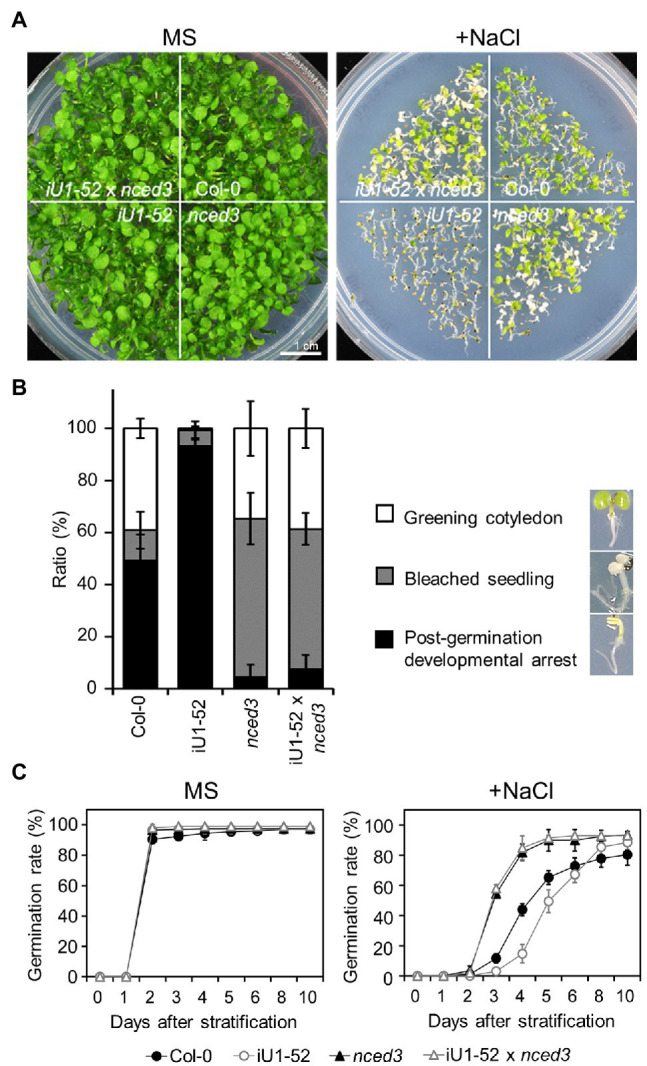
The *nced3* mutant allele abrogates salt hypersensitivity in iU1 plants **(A)**. Comparison of the seedling phenotypes. The seedlings were grown on agar plates supplemented with or without NaCl (200 mM) for 14 days. **(B)** Phenotypic quantification derived from **(A)**. **(C)** Seed germination rate. The seeds were sown on agar plates supplemented with or without 200 mM NaCl. Data are presented as the means ± SD of at least three biological replicates. Each replicate contained ~66 seeds.

To further verify whether the salt hypersensitivity of the iU1s is the consequence of the elevated ABA levels, fluridone, an ABA biosynthesis inhibitor, was applied in salt-containing media. The results indicated that fluridone rescued both the germination delay (Supplementary Figures S7A–F) and postgermination developmental arrest (Supplementary Figures S7C,G) in iU1s under salt-stressed conditions. Fluridone is a systemic herbicide that inhibits carotenoid biosynthesis and leads to the interruption of chlorophyll and ABA biosynthesis (Bartels and Watson, 1978). Thus, the fluridone-treated seedlings exhibited an albino-like phenotype (Supplementary Figure S7C). Furthermore, the ABA-deficient mutant allele, *9-cis-epoxycarotenoid dioxygenase 3* (*nced3*), which is involved in the ABA biosynthetic pathway, was introduced into the iU1-52 plants through the crossing. The *nced3* mutant alleles abrogated the low seed germination rate (Figures 7A,C) and postgermination developmental arrest (Figure 7B) of iU1-52 under salt stress. As ABA is a plant stress hormone, ABA deficit in *nced3* and iU1-52 x *nced3* resulted in an increase in bleached cotyledons under a salt treatment for 14 days. These data provide evidence that the salt hypersensitivity of iU1s is dependent on ABA levels.

### *GlcNA.UT*-Mediated Gene Expression Profiling

**Figure 8:**
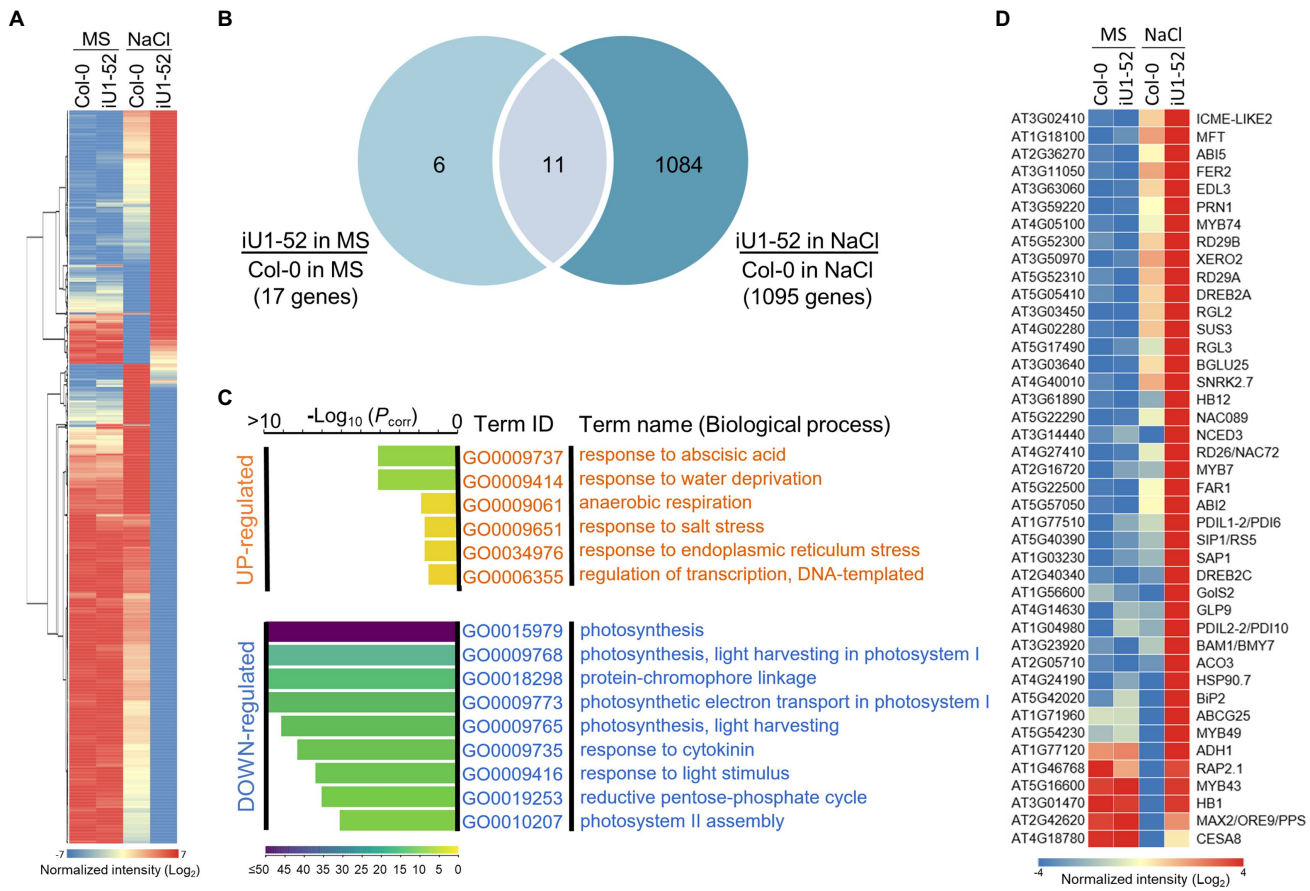
*GlcNA.UT*-mediated gene expression profiles. **(A)** Heatmap of differentially expressed gene (DEG) profile. Total RNA was derived from seedlings grown on agar plates supplemented with or without 200 mM NaCl for 14 days. A total of 1,101 DEG genes derived from the microarray assay were used in this analysis. **(B)** Venn diagram indicating the overlap of DEG genes between salt and salt-free treatments. **(C)** Gene ontology (GO) analysis of biological processes. **(D)** Heatmap showing DEG genes involved in ABA, water deprivation, salt and ER stress responses. For the microarray assay, three biological replicates were performed, and the raw data are available in the Gene Expression Omnibus (GEO) database, with accession No. GSE193841.

To further better understand the *GlcNA.UT*-mediated downstream gene expression profiles, analyses of Arabidopsis gene expression microarray (Agilent) were performed. The samples used in this assay were derived from the wild-type (Col-0) and iU1-52 seedlings grown on MS or MS + NaCl (200 mM) agar plates for 14 days. Differentially expressed gene (DEG) were defined by the gene signal (iU1-52 normalized to Col-0) over two-fold (≥2 or ≤ −2). Based on these criteria, the majority of gene expression under normal growth conditions displayed a similar pattern between iU1-52 and Col-0 except that 17 DEGs were observed (Figures 8A,B); among them, 6 genes were upregulated and were specific to normal growth conditions (Supplementary Figure S8B). These genes have functions in defense response (*DEFL* and *pectate lyase*), GPI-anchored protein (*LTPG17*), and sterol transport (At5g23840 and At5g23830). Eleven genes overlapped between salt-free and salt-stressed conditions (Supplementary Figure S8A). These genes are primarily involved in defense responses (such as *TRAF-like protein*, *MBP1*, *WRKY38*, *PR2* and *VSP2*), together with iron ion transport [such as *IRONMAN 1 (IMA1*) and *IMA2*], response to heat stress (*TMS1*) and ER stress (*TIN1*), and metabolic processes [*GlcNA.UT1* and *FAD/NAD(P) binding oxidoreductase*]. Consistent with the function of RNA interference, the expression of *GlcNA.UT1* was significantly reduced in iU1-52 under normal or salt-stressed conditions. It is surprising that the gene expression of *GlcNA.UT2* was not significantly reduced in iU1-52, in which *GlcNA.UT2* lost its function with undetectable protein levels (Figure 1E). This is most likely because the transcript of *glcna.ut2-1* is truncated, and its transcript is steadily detectable (Chen et al., 2014) and can be recognized by microarray probes. In contrast, ~1,095 DEGs were observed in the iU1-52 seedlings grown under salt stress (Figures 8A,B). Among them, 1,084 DEGs (375 upregulated and 709 downregulated), which were specifically shown in salt-treated iU1-52 seedlings, were further analyzed through the Gene Ontology (GO) of biological processes. For upregulated DEGs, several biological processes were overrepresented, such as the response to ABA, water deprivation, hypoxia, salt stress, ER stress, and transcription regulation. However, the top nine biological processes from downregulated DEGs were enriched in photosynthesis-related functions (Figure 8C), which support the iU1 phenotype showing post-germination developmental arrest under salt stress. At least 42 DEGs involved in the ABA response, water deprivation, salt stress and ER stress are listed (Figure 8D). These genes included ABA biosynthesis, transport and signaling (such as *NCED3*, *ABI2*, *ABI5*, *SnRK2* and *ABCG25*), salt stress (such as *RD29A*, *RD29B*, *BGLU25* and *ATHB1*), and ER stress (such as *PDIL* and *BIP2*), which were upregulated in iU1-52 seedlings under salt stress. Some of these genes were verified by RT-qPCR and showed similar expression patterns to that of the microarray data (Supplementary Figure S9). All the mutants used in this study, such as iU1s, were verified by genotyping (Supplementary Figure S10). These data further support that the sensitivity of iU1s to salt is primarily associated with ABA biosynthesis and its signaling networks.

## Discussion

### *Glcna.UT*s Are Necessary for UDP-GlcNAc Biosynthesis and Protein N-Glycosylation

GlcNAc is the fundamental amino sugar moiety essential for protein glycosylation and sugar conjugates of lipids and proteins (Pattison and Amtmann, 2009; Nagashima et al., 2018). Completely blocking the HBP pathway often results in lethality in plants (Riegler et al., 2012; Chen et al., 2014; Vu et al., 2019), bacteria (Mengin-Lecreulx and van Heijenoort, 1993), yeast (Mio et al., 1998), *Caenorhabditis elegans* (Denzel et al., 2014), and *Drosophila melanogaster* (Schimmelpfeng et al., 2006), reflecting the vital role of GlcNAc in the normal growth of organisms. For organisms that possess lethal phenotypes due to a loss in function of HBP biosynthetic genes, RNA interference of these genes was frequently used to study their functions in plants (in this study; Vu et al., 2019) and in *C. elegans* (Denzel et al., 2014). The *Arabidopsis* genome contains two *GlcNA.UT*s, i.e., *GlcNA.UT1* and *GlcNA.UT2*, which encode GlcNAc1pUT1 and GlcNAc1pUT2, respectively (Yang et al., 2010). Likewise, the rice genome also contains two *GlcNA.UT*s, which are referred to as *UAP1/SPL29* and *UAP2* (Wang et al., 2015, 2021). In general, both proteins in *Arabidopsis* and rice have a catalytic ability to utilize GlcNAc-1-P and GalNAc-1-P as substrates to form UDP-GlcNAc (Yang et al., 2010; Xiao et al., 2018; Wang et al., 2021). In addition, Arabidopsis GlcNAc1pUT2 may also use Glc-1-P as a substrate to form its corresponding UDP-sugars (Yang et al., 2010). In rice, OsUAP1/SPL29-1 irreversibly catalyzes the decomposition of uridine 5′-diphosphoglucose-glucose (UDPG). The *Osuap1*/*spl29* mutants lose enzymatic activity and result in the accumulation of UDPG, which is implied as an important component involved in ROS accumulation, programmed cell death and leaf lesion mimics (Xiao et al., 2018). It remains unknown whether Arabidopsis GlcNAc1pUTs may use UDPG as a substrate. Interestingly, no conceivable phenotype was observed for the single knockout mutant of Arabidopsis *glcna.ut1* or *glcna.ut2* (this study; Chen et al., 2014), whereas early leaf senescence and defense-response-related lesion-mimic spotted leaves are observed in a single mutant of rice *Osuap1*/*spl29* (Wang et al., 2015, 2021; Xiao et al., 2018). As the expression of defense-responsive genes was also altered in iU1s (Supplementary Figure S8), this subtle discrepancy in leaf lesion mimics between rice and *Arabidopsis* reveals the possibility that rice leaves have a greater sensitivity to defense responses than that of *Arabidopsis* leaves. Further investigation is necessary to determine this discrepancy.

Although both AtGlcNAc1pUTs and OsUAPs have the enzymatic activity to form UDP-GlcNAc, their biochemical effects on UDP-GlcNAc biosynthesis in planta and on protein N-glycosylation are not yet illustrated. It has been reported that compared to UDP-GalNAc, UDP-GlcNAc is the major component of UDP-HexNAc in *Arabidopsis*, and the ratio of UDP-GlcNAc/GalNAc is maintained in the wild type under normal or high-temperature conditions (Nozaki et al., 2012). It is also implied that epimerization might occur on GlcNAc or GalNAc-derived metabolites in the HBP (Nozaki et al., 2012) because plants possess epimerases that are capable of converting the Glc-moiety of sugar derivatives to the Gal-moiety conjugates (Allard et al., 2001). In this study, we demonstrated that the Arabidopsis UDP-HexNAc (UDP-GlcNAc and UDP-GalNAc) contents were similar between the wild-type and iU1 seedlings under normal growth conditions. This is probably because the residual GlcNAc1pUT1 activity in iU1s is capable of producing a sufficient amount of UDP-HexNAc. In contrast, the UDP-HexNAc contents were lower in iU1 seedlings than those in wild-type seedlings under salt-stressed conditions (Figure 3), indicating that the residual activity of GlcNAc1pUT1 in iU1s did not effectively generate a sufficient amount of UDP-HexNAc in response to salt stress. Consistently, the exogenous GlcNAc enhances UDP-HexNAc biosynthesis under normal growth conditions in both wild-type and iU1 plants (this study; Furo et al., 2015). This supports that GlcNAc can be converted to GlcNAc-6-P by GNK through the salvage pathway, and then, GlcNAc-6-P enters the HBP pathway for UDP-GlcNAc biosynthesis (Supplementary Figure S1). However, under salt-stressed conditions, GlcNAc application enhances UDP-HexNAc biosynthesis exclusively in the wild-type seedlings but not in iU1s (Figure 3), presumably due to the defect in GlcNAc1pUT1 that fails to effectively produce UDP-HexNAc from GlcNAc-1-P. The mechanism by which exogenous GlcNAc improves salt stress tolerance (Figure 3) is likely because the increase in UDP-GlcNAc levels in the wild-type seedlings enhances the capacity of protein glycosylation and attenuates ER stress. In parallel to the normal levels of UDP-HexNAc under normal growth conditions, the pattern of N-glycosylation proteins was also comparable among wild-type, single mutants and iU1 seedlings. In contrast, N-glycosylation patterns were altered in iU1 seedlings compared to wild-type and single mutants under salt stress, based on complex N-glycan assays by ConA and anti-HRP antibody or by detecting specific N-glycoproteins, PDI5 and TGGs (Figure 4). Collectively, our data provided evidence that GlcNAc1pUTs play a vital role in UDP-GlcNAc biosynthesis and protein N-glycosylation, particularly under salt-stressed conditions.

### Salt Sensitivity in iU1s Is Distinct From the Mutants Defective in N-Glycan Processing in the ER Lumen or Golgi Apparatus

GlcNAc is essential for N-glycan processing and protein N-glycosylation. Interruption of any step during N-glycan processing or protein N-glycosylation will cause an accumulation of unfolded or misfolded proteins in the ER lumen, resulting in ER stress. Subsequently, ER stress induces an unfolded protein response (UPR) by triggering the expression of genes involved in enhancing the capacity of protein folding, increasing the ER quality control, and maintaining ER homeostasis (Bao and Howell, 2017; Nagashima et al., 2018). Previous studies have indicated that the UPR plays an important role in the response to abiotic stress, such as salt stress (Koiwa et al., 2003; Frank et al., 2008; Kang et al., 2008; Zhang et al., 2009). For example, N-glycan processing mutants, such as *staurosporine* and *temperature sensitive 3a* (*stt3a*) and *leaf wilting 3* (*lew3*), which are defective in a catalytic subunit of the ER oligosaccharyltransferase (OST) complex and in an α-1,2-mannosyltransferase, respectively, induce UPR-mediated gene expression, such as *BiP*s, and enhance salt stress sensitivity (Koiwa et al., 2003; Zhang et al., 2009). Moreover, transgenic plants overexpressing the UPR-responsive gene *BiP* in tobacco or soybean confer tolerance to ER stress and drought (Alvim et al., 2001; Valente et al., 2009). These data indicate that the UPR-mediated pathway interplays with osmotic stress signaling pathways.

*stt3a* causes defects in protein N-glycosylation, which induces UPR in the ER and enhances salt sensitivity (Kang et al., 2008). Similarly, the *cgl1* mutant, which lacks N-ACETYL GLUCOSAMINYL TRANSFERASE I activity, shows a deprivation of complex N-glycans and confers salt oversensitivity (Frank et al., 2008). However, unlike *stt3a*, *cgl1* does not exhibit a UPR response because it fails to activate the expression of *BiP3*, a UPR marker gene. These data indicate that UPR-responsive signaling is not the major mechanism that leads to salt oversensitivity in *stt3a*. Thus, Kang et al. (2008) proposed that the maturation of N-glycosylated proteins in the Golgi apparatus is essential for salt tolerance. However, mutation of *UDP-GlcNAc transporter 1* (*UGNT1*), which is localized in the Golgi membrane and deprived of complex and hybrid N-glycans in the Golgi, does not result in salt oversensitivity, reflecting that mature complex N-glycans are not the only primary factor in response to salt sensitivity (Ebert et al., 2018). The sites of complex N-glycan attachment within a glycoprotein and additional mechanisms cooperate to make glycoproteins functional (Rips et al., 2014). It was proposed that each mutant defective in N-glycan processing might affect a different set of glycoprotein and/or glycolipid functions, in which the signals integrate to alter plant growth and response to abiotic stress. For instance, the *stt3a* mutant exhibits mitosis arrest in the root apical meristem and radial swelling in the root tips and alters the abundance and function of *KORRIGAN1* (*KOR1*) /*RADIALLY SWOLLEN 2* (*RSW2*), an endo-ß-1,4-endoglucanase activity involved in cell wall biosynthesis (Kang et al., 2008; Mansoori et al., 2014; Vain et al., 2014). The *mns1* and *mns2* double mutants, which lack α-1,2 mannosidase activity, exhibit severe inhibition of root growth under salt stress and defects in cell wall biosynthesis, which is mediated by *KOR1*/*RSW2*. These salt-sensitive root phenotypes can be partially rescued by the overexpression of *KOR1*/*RSW2* in this double mutant, indicating that trimming of N-glycans is crucial in mediating its targeted glycoprotein abundance to adapt to salt stress (Liu et al., 2018). Consistently, the *kor1*/*rsw2* mutant is defective in root growth, and the correct destination of KOR1/RSW2 in the plasma membrane is associated with salt acclimation (Liu et al., 2018; Nagashima et al., 2020). When comparing iU1s with *stt3a*, both revealed defects in protein N-glycosylation and induction of the UPR response. However, unlike *stt3a*, which possessed a short root length under salt stress (Koiwa et al., 2003), iU1 displayed normal root elongation under salt-stressed growth conditions (Supplementary Figure S2). iU1s that exhibited salt oversensitivity were based on seed germination delay and postgermination developmental arrest; instead, these two phenotypes were almost normal in *stt3a* (Supplementary Figure S2). Thus, it is likely that iU1 and *stt3a* use different mechanisms to enhance salt sensitivity. It is notable that the UDP-GlcNAc, the product of the GlcNAc1pUT catalytic reaction, is not only used for N-glycan synthesis and maturation in the ER or Golgi apparatus (Ebert et al., 2018) but also for the O-linked GlcNAcylation of cytosolic and nuclear proteins, and sugar moieties of glycolipids and GPI-anchored proteins. Thus, it is conceivable that although both iU1 and *stt3a* exhibited salt oversensitivity, they displayed different salt-responsive phenotypes.

The ER stress-induced UPR response has been proposed primarily through pathways that are mediated by IRE1-bZIP60 and S1P-bZIP17/or S2P-bZIP28, which are linked to abiotic stress responses and the transport and sensing of phytohormones, such as auxin and brassinosteroid (Fujita et al., 2007; Liu et al., 2007a; Che et al., 2010; Liu and Howell, 2010; Chen et al., 2014; Bao and Howell, 2017; Iwata et al., 2017). The *atbzip17* mutant displays salt sensitivity, and this can be complemented by the overexpression of At*bZIP17* gene. However, transgenic plants overexpressing *35S::AtbZIP17∆*, a truncated gene without transmembrane and lumen-facing domains, display retarded phenotypes, such as a small size and short roots and hypocotyls, under stress-free conditions. Moreover, the retarded phenotypes can be rescued by the expression of *AtbZIP17∆* driven by the *RD29A* promoter, and transgenic plants enhance salt tolerance, based on the bleached seedlings observed under agar plates and soil-growth conditions (Liu et al., 2007b, 2008). Although the AtbZIP17-activated domain induces the expression of ER stress response genes, such as *AtHB7*, *RD20*, and *PP2C*, the ER marker genes *BiP1*, *BiP2* and *BiP3* are not upregulated (Liu et al., 2007b). In contrast, the expression of *BiP*s is often upregulated in the mutants involved in N-glycan processing, such as iU1s, *stt3a*, *lew3*, and *lew1* (this study; Koiwa et al., 2003; Zhang et al., 2008, 2009; Nozaki et al., 2012). Moreover, the expression of *IRE1*, *S1P*, *S2P*, *bZIP17*, *bZIP28*, and *bZIP60* was not significantly induced in our transcriptomic analyses. In addition, although mutants with altered N-glycosylation often enhance the sensitivity of Tm (this study; Zhang et al., 2008, 2009), Tm treatments with wild type and iU1s did not induce salt oversensitive phenotypes in terms of seed germination delay and severe postgermination developmental arrest (Supplementary Figure S6). These data indicated that the response of iU1 to the Tm treatment is different from its response to salt-stressed conditions. Thus, the induction of UPR-responsive gene expression by Arabidopsis activated forms (or N-termini) of bZIP17, bZIP28, or bZIP60 (Liu et al., 2007a,b; Bao and Howell, 2017) or by N-glycan processing mutants, such as iU1s (in this study) and *stt3a*, might use, at least in part, distinct regulatory signaling pathways.

### Salt Sensitivity in iU1s Is Associated With ABA Biosynthesis and Signaling

The plant stress hormone ABA plays a major role in normal plant growth and facilitates the adaptation of plants to abiotic stress (Finkelstein et al., 2002). The *LEW3* functions in transferring mannose to the dolichol-linked oligosaccharide in the last two steps on the cytosolic side of the ER (Zhang et al., 2009). Mutation of *LEW3* in *Arabidopsis* enhances salt and ABA sensitivity compared to that of the wild-type plants. Under salt stress conditions, seed germination and the establishment of early seedlings with greening cotyledons are much lower in *lew3* seedlings than in the wild-type seedlings. Although these phenotypes resemble iU1s, the *lew3* mutants grown in soil display small plant sizes and tend to wilt, which is presumably due to the collapse of xylems and interruption of water transport (Zhang et al., 2009). The *LEW2* gene, also known as *AtCesA8*/*IRX1*, encodes a subunit of the cellulose synthesis complex. Mutation of *LEW2* causes small plant sizes under normal soil-growth conditions. Moreover, the *lew2* mutants contain higher levels of ABA, proline and sugars, in association with higher expression of *ABA2*/*SDR1*, *P5CS* (pyrroline-5-carboxylate synthase), and RD29A (Chen et al., 2005) under normal and osmotic stress conditions. However, unlike *lew3* showing seed germination delay, the *lew2* mutant promotes seed germination under osmotic stress, including salt stress. These data provide evidence that cellulose synthesis is important for the osmotic stress response. ABA accumulation in plants suffering from abiotic stress is mediated primarily by a multistep *de novo* biosynthesis pathway or by single-step hydrolysis of inactive ABA-glucose ester (ABA-GE; Nambara and Marion-Poll, 2005). Hydrolysis of ABA-GE is catalyzed by ER-localized ß-glucosidase 1 (BG1/BGLU18) or vacuole-localized BG2/BGLU33. The ER dynamics are vital processes when plants face stress environments. One of the most obvious phenomena is the dynamic change of ER bodies in response to environmental stress. BG1/BGLU18 is the main component of ER body and its hydrolysis of ABA-GE may cause early accumulation of ABA that precedes the *de novo* biosynthesis pathway (Lee et al., 2006; Xu et al., 2012; Han et al., 2020). The *stt3a* mutant showed salt sensitivity and defect in protein N-glycosylation (Kang et al., 2008). Further studies indicated that *stt3a* reveals a high transpiration rate, sensitivity to drought stress and abnormal stomatal distribution. Moreover, these phenotypes are associated with the decrease in ABA and IAA, and exogenous ABA and IAA may partially rescue these mutant phenotypes. The lower levels of ABA in *stt3a* are linked to the underglycosylation of BG1/BGLU18 (Jiao et al., 2020). Consistently, the transcriptomic analyses indicated that the expression of *BG1*/*BGLU18* in iU1 was ~2.9-fold lower than the wild type, but the expression of *BGLU25* in iU1 was ~2.8-fold higher than the wild type under salt stress conditions (Figure 8D). These data support that N-glycosylation is involved in abiotic stress response through the release of ABA from ABA-GE hydrolysis. Given that BG1/BGLU18 primarily functions in ABA release in plants exposed to a short-term stress treatment, the effect of this protein and/or BGLU25 on ABA release is likely minor in iU1s because a long-term salt treatment up to 14 days was performed in this study, and the expression of *NCED3*, a key gene in ABA *de novo* biosynthesis, was upregulated. In addition, ABA-responsive genes, such as *ABCG25*, *ABI2*, *ABI5*, and *RD29A*, and other salt-responsive genes, such as *RD29B*, *SNRK2.7*, *HB12*, and *FAR1*, were also upregulated (Figure 8; Supplementary Figure S9). Moreover, the iU1 seedlings enhanced ABA sensitivity, which showed a phenotype similar to salt hypersensitivity (Figures 6A,B). In addition, salt-sensitive phenotypes could be eliminated by the exogenous ABA biosynthesis inhibitor fluridone and the introduction of a *necd3* allele into the iU1 plants (Figure 7; Supplementary Figure S7). Thus, these data provide evidence that *GlcNA.UT*s play an important role in response to salt stress through ABA biosynthesis and signaling networks, which are different from the known mutants of N-glycan processing. Currently, the mechanism by which the reduced UDP-GlcNAc levels interplay with ABA levels and signaling largely remains unknown. One possibility is that the reduced UDP-GlcNAc levels under salt-stressed conditions alter specific N- and/or O-linked glycoproteins that further regulate ABA biosynthesis and signaling. Thus, further investigation is essential to define these ABA-related glycoproteins in the future.

ER-associated degradation (ERAD) is one of the UPR responses, which mediates the protein quality control to release ER stress and maintain ER homeostasis. One of the major processes in ERAD is the binding of misfolded proteins by the Hrd1 complex to facilitate the ubiquitination of these defective proteins. Subsequently, these ubiquitinated proteins are retrotranslocated into the cytosol for proteasomal degradation. In this study, the expression of *Hrd1B*, which encodes a component of the Hrd1 complex, showed an ~2.3-fold increase in iU1s under salt stress. Moreover, ABA INSENSITIVE RING PROTEIN 2 (AIRP2), encoding a cytosolic RING-type E3 ubiquitin ligase, was also with induced by approximately 3-fold under salt stress conditions. It has been proposed that AIRP2 and SALT- AND DROUGHT-INDUCED RING FINGER1 (SDIR1), an ER E3 ligase, likely play a combinatory role in ABA signaling and the response to high salt in *Arabidopsis* (Oh et al., 2017). Thus, these data support the possible involvement of ERAD in salt stress response in iU1s. Furthermore, the salt-treated iU1 seedlings also increased the expression of *protein disulfide isomerase*s, such as *PDI6* and *PDI10*, and *heat shock protein*s, such as *HSP90.7* and *BiP2* (Figures 5, 8), which are able to enhance protein folding capacity and release ER stress. Collectively, these data provide evidence that *GlcNAc.UT*s integrate multiple regulatory pathways into the adaptation of plants to salt stress through protein glycosylation, ER dynamics (protein quality control and protein folding) and ABA synthesis and signaling.

## Conclusion

Despite the significance of protein glycosylation in plant growth and abiotic stress responses, most studies in the past have extensively focused on the formation of core N-glycans in the ER lumen and the modification of complex N-glycans in the Golgi apparatus. Although functions of HBP-related genes have been reported to have functions in plant growth and development, the effect of GlcNAc on the cytosolic processing of oligosaccharide precursors for protein glycosylation and abiotic stress response is less clear, particularly for *GlcNA.UT*s. In this study, we demonstrated that *GlcNA.UT*s play vital roles in UDP-GlcNAc biosynthesis, N-glycosylation and ABA-mediated salt stress responses under salt-stressed conditions. Moreover, *GlcNA.UT*s also affect the expression of O-GlcNAcylated protein genes, which remain to be further investigated regarding their functions in plant growth and salt stress responses.

## Data Availability Statement

The datasets presented in this study can be found in online repositories. The names of the repository/repositories and accession number(s) can be found in the article/Supplementary Material.

## Author Contributions

Y-HC performed most of the experiments. H-LS, S-JC, and YS performed some of the experiments. W-HC conceived the original research plans and supervised the experiments. Y-HC and W-HC wrote the article with help from all authors. All authors contributed to the article and approved the submitted version

## Funding

This work was supported by the Ministry of Science and Technology (MOST), Taipei, Taiwan (MOST-104-2311-B-001-024; MOST-105-2311-B-001-073).

## Conflict of Interest

The authors declare that the research was conducted in the absence of any commercial or financial relationships that could be construed as a potential conflict of interest.

## Publisher’s Note

All claims expressed in this article are solely those of the authors and do not necessarily represent those of their affiliated organizations, or those of the publisher, the editors and the reviewers. Any product that may be evaluated in this article, or claim that may be made by its manufacturer, is not guaranteed or endorsed by the publisher.
