# Supplementary material for: Interference of Arabidopsis N-Acetylglucosamine-1-P Uridylyltransferase Expression Impairs Protein N-Glycosylation and Induces ABA-Mediated Salt Sensitivity During Seed Germination and Early Seedling Development
Source: Front Plant Sci. 2022 Jun 7;13:903272. doi: 10.3389/fpls.2022.903272 (PMC9210984; doi:10.3389/fpls.2022.903272)
Supplement: Supplementary file 1 [file Data_Sheet_1.pdf]

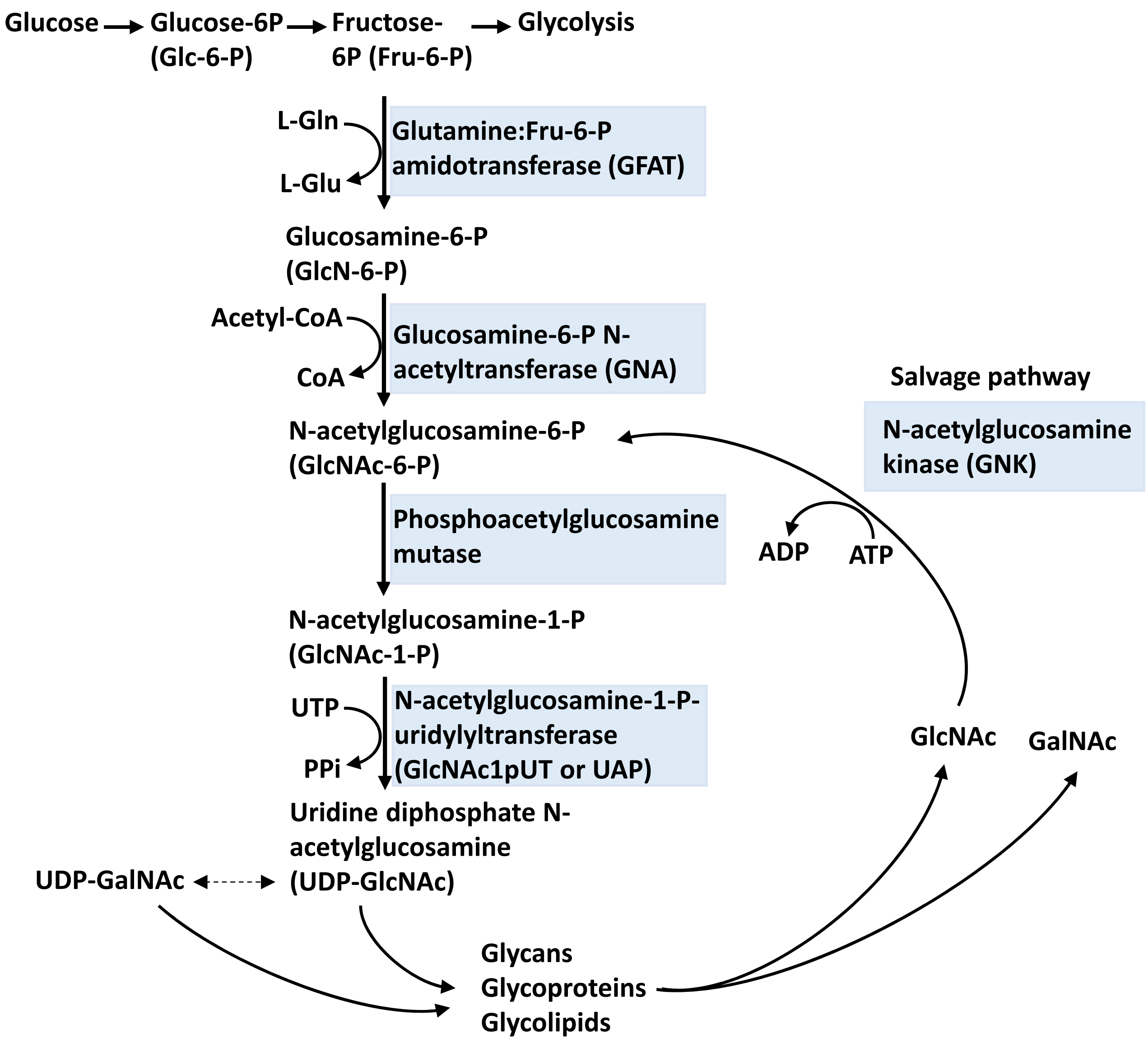

Supplementary Figure S1. Schematic diagram showing UDP-GlcNAc biosynthesis through the hexosamine biosynthetic pathway. The hexosamine biosynthetic pathway (HBP) includes four-step reactions, which are catalyzed orderly by glutamine:Fru-6-P amidotransferase (GFAT), glucosamine-6-P N-acetyltransferase (GNA), phosphoacetylglucosamine mutase and N-acetylglucosamine-1-P uridylyltransferase (GlcNAc1pUT or UAP) to synthesize uridine diphosphate N-acetylglucosamine (UDP-GlcNAc). UDP-GlcNAc is presumably converted to UDP-N-acetylgalactosamine (UDP-GalNAc) by an unidentified epimerase in plants. N-acetylglucosamine kinase (GNK), a key enzyme in the salvage pathway, uses GlcNAc as a substrate to produce GlcNAc-6-P that enters the HBP for UDP-GlcNAc biosynthesis. This diagram is modified from Furo et al., 2015.

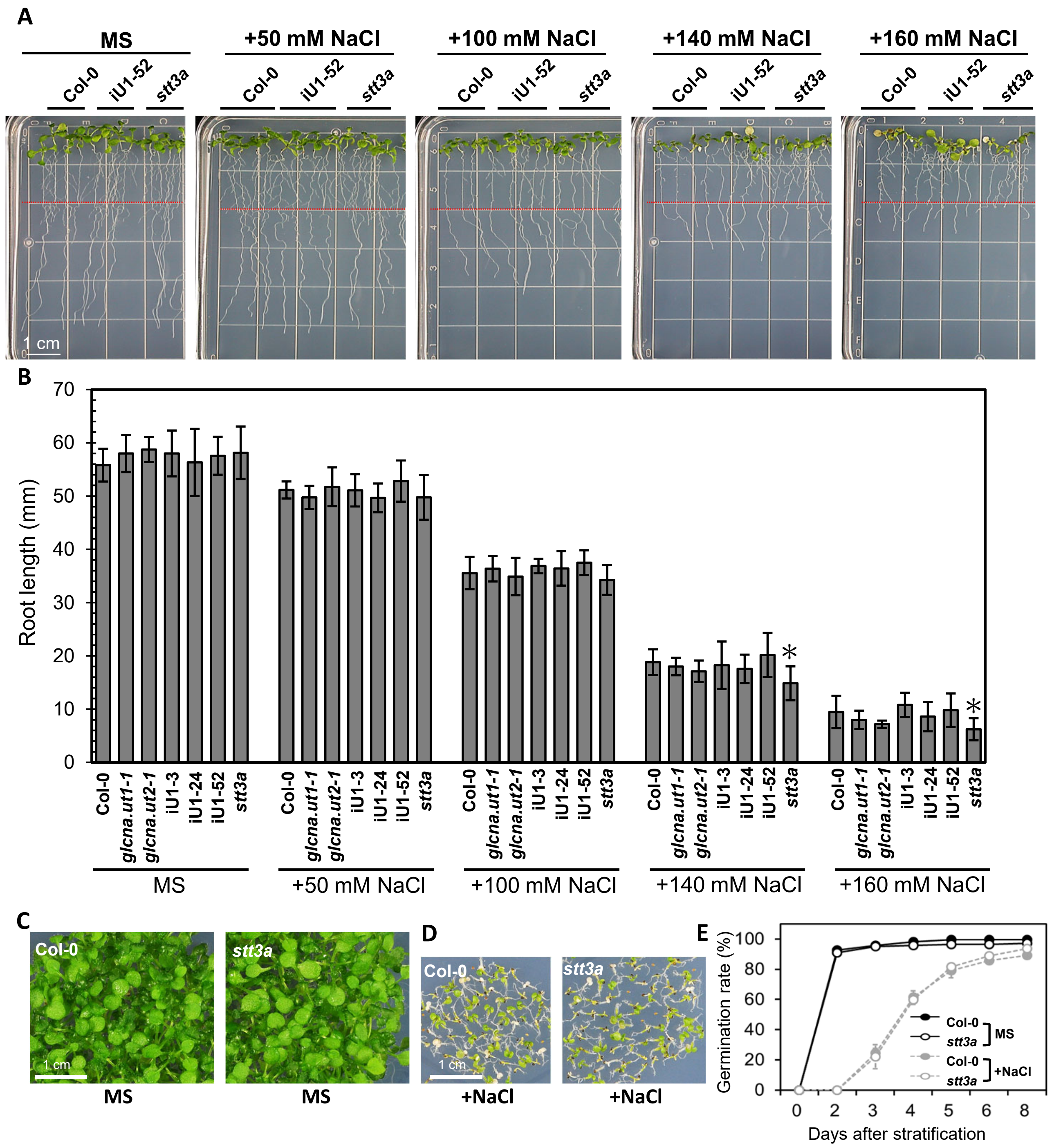

Supplementary Figure S2. Effect of salt stress on root growth. **(A)** Comparison of root growth under treatments with different salt concentrations. Seedlings were grown on MS media for 6 d and then transferred to fresh media supplemented with or without salt stress for another 4 days. The red dashed lines indicate the starting point of root growth after seedling transfer. **(B)** Quantification of the root length derived from (A). The values in (B) indicate the means ± SD of eight primary root lengths of seedlings. Two independent experiments were performed, and consistent results were obtained. *, p<0.05, Student's t-test. **(C, D)** Comparison of phenotypes. Seedlings were grown on agar plates supplemented without (C) or with 200 mM NaCl (D) for 14 days. **(E)** Seed germination rate. Seed germination was counted, based on the experiments shown in (C, D). The value represents the means ± SD of three independent experiments, each with approximately 60 seeds.

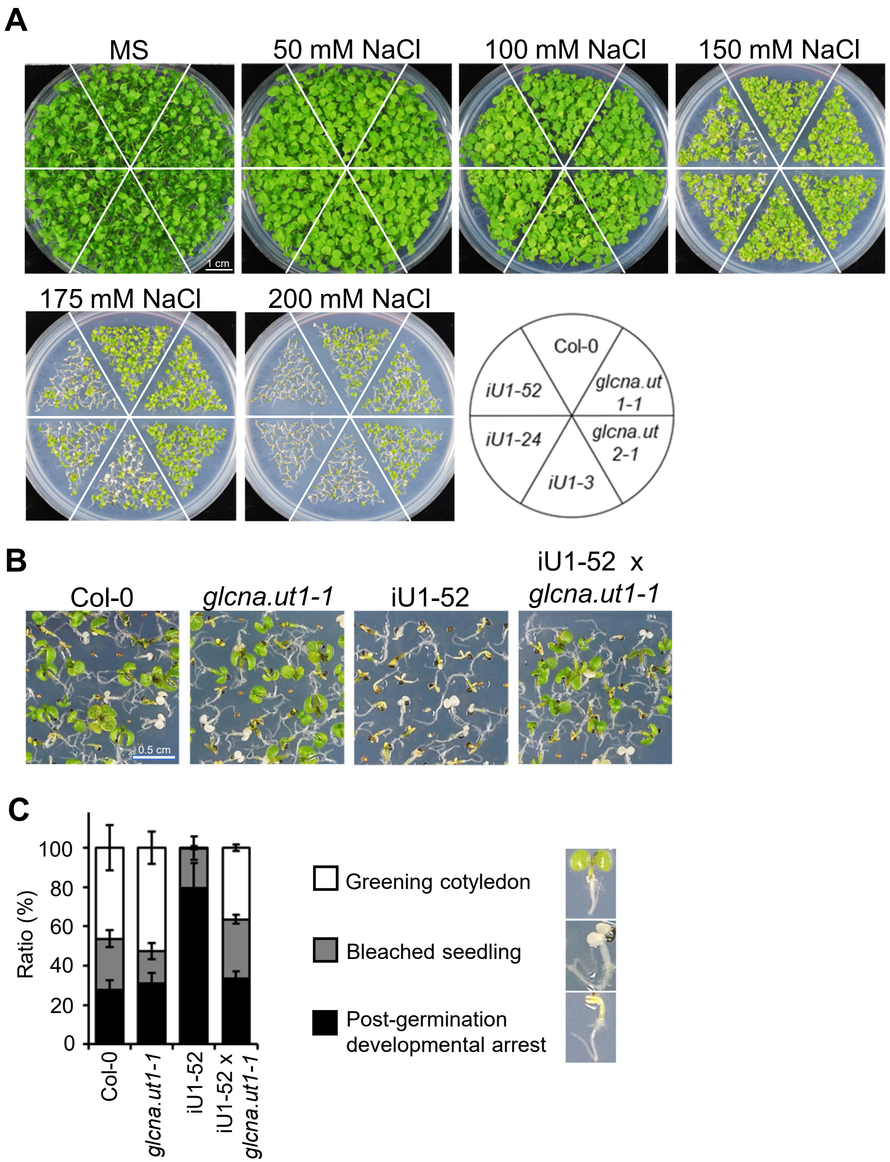

Supplementary Figure S3. Salt hypersensitivity of the iU1 lines. **(A)** Phenotypic comparison. Seedlings were grown on agar plates supplemented with different salt concentrations for 14 days. **(B)** RNAi off-target examination. Seedlings were grown on agar plates for 14 days. **(C)** Quantification of the seedling phenotypes derived from (B). The value represents the means ± SD of three independent experiments, each with approximately 60 seeds.

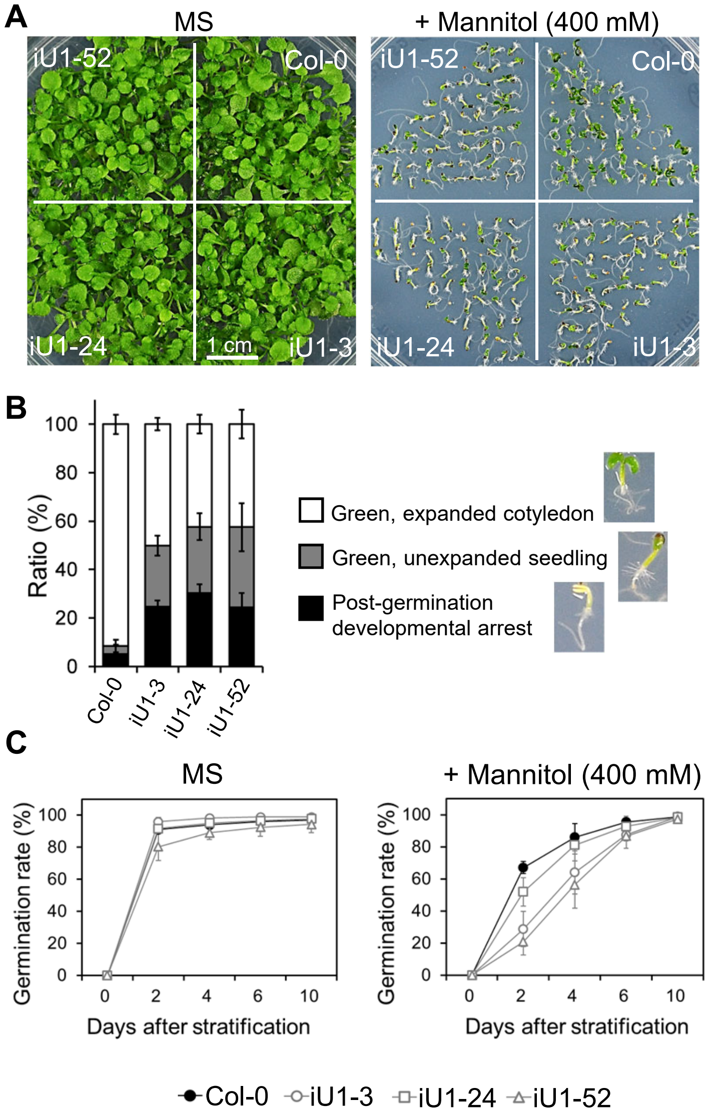

Supplementary Figure S4. Effect of osmotic stress on seed germination and the establishment of early seedlings. **(A)** Phenotypic comparison. Seedlings were grown on agar plates supplemented with or without 400 mM mannitol. **(B)** Quantification of the seedling phenotypes derived from (A). **(C)** Seed germination rate. Seeds were grown on agar plates supplemented with or without 400 mM mannitol for a period of time as listed. Data represent the means ± SD of four independent experiments, each with approximately 60 seeds.

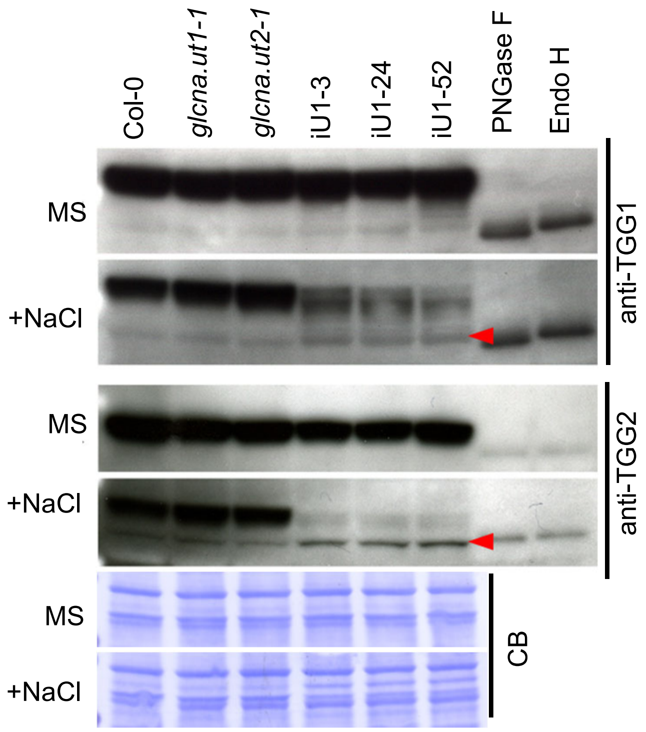

Supplementary Figure S5. Impairment of TGG protein N-glycosylation under salt stress. Seeds were grown on agar plates supplemented with or without salt (200 mM NaCl) for 14 days. Subsequently, the seedlings were used for protein extraction and protein blot analyses. The red arrowheads represent unglycosylated TGG proteins. Coomassie blue (CB) staining was used as a loading control.

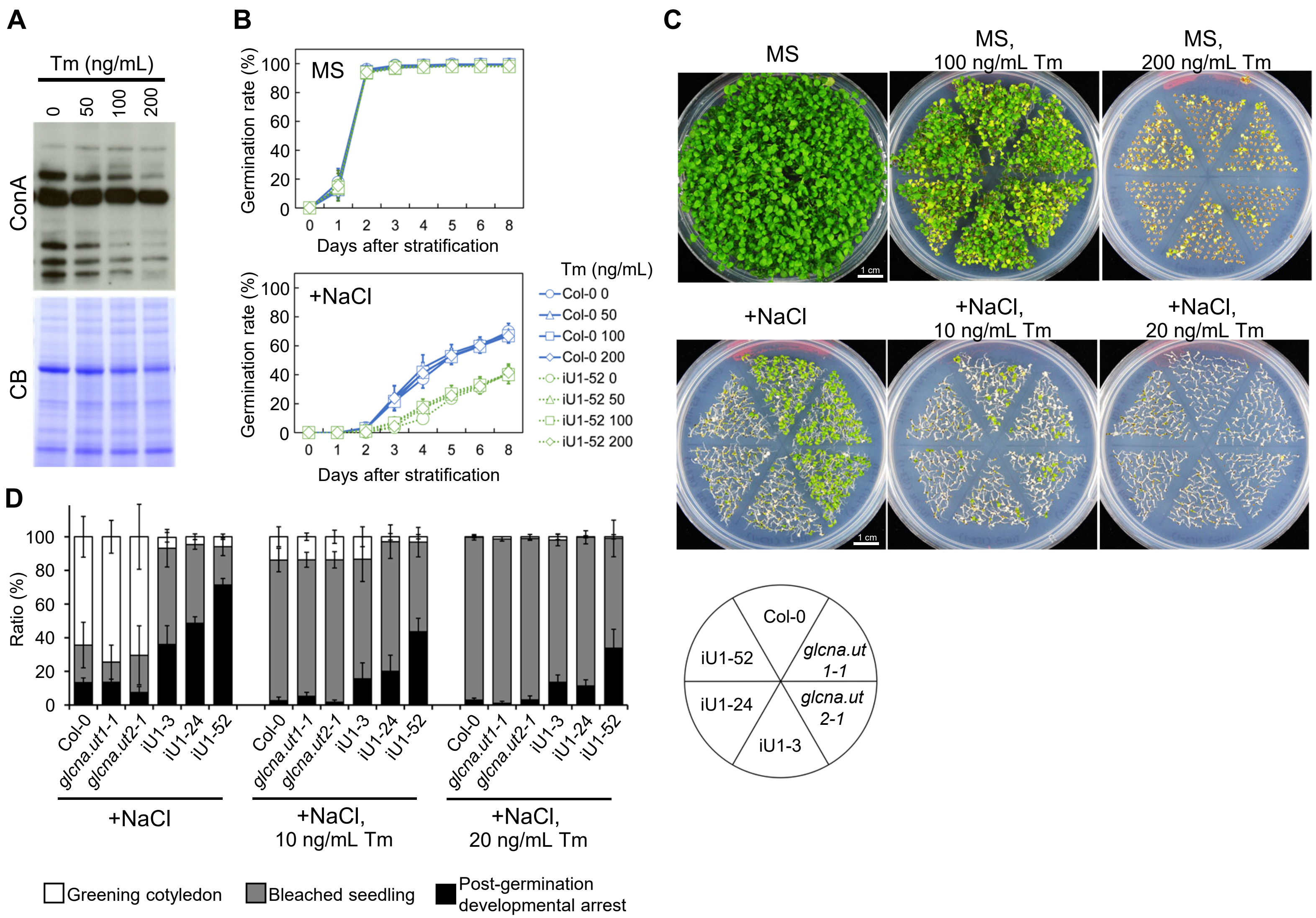

Supplementary Figure S6. Effect of tunicamycin (Tm) on protein N-glycosylation, seed germination and the establishment of early seedlings. **(A)** Tm treatment. Seeds were grown on agar plates supplemented with different concentrations of Tm for 14 days, followed by protein extraction and protein blot analysis. Coomassie blue (CB) staining represents the loading control. **(B)** Seed germination rate. Seeds were grown on agar plates supplemented with or without Tm for a period of time as listed. **(C)** Phenotypic comparison of seedlings under Tm and/or salt treatment. Seeds were grown on agar plates supplemented with Tm and/or salt (200 mM NaCl) for 14 days. **(D)** Quantification of seedling phenotypes. The values represent the means ± SD of four biological repeats, each with approximately 45 seeds.

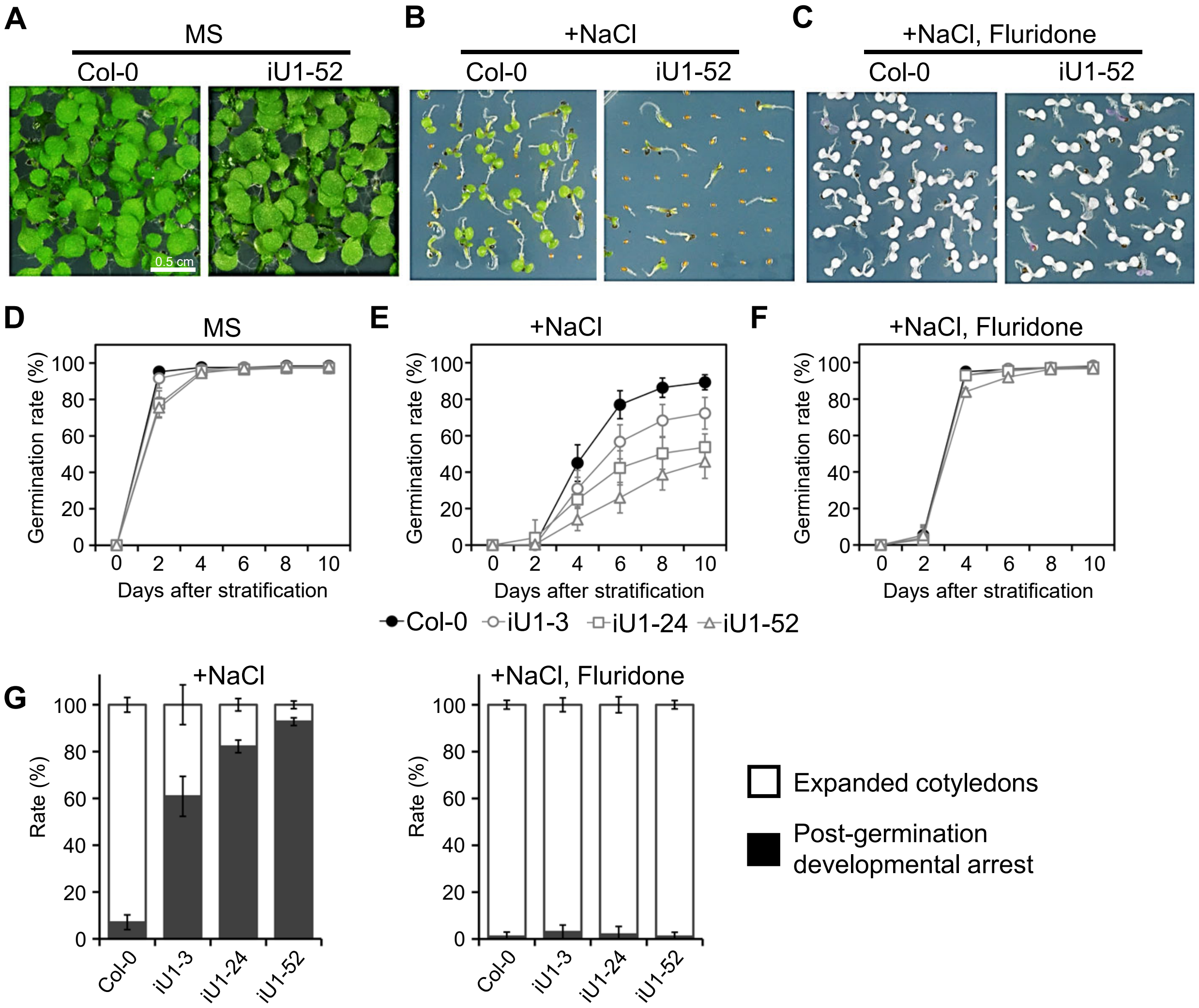

Supplementary Figure S7. ABA biosynthesis inhibitor fluridone rescues salt hypersensitivity of iU1 seedlings. **(A-C)** Comparison of seedling phenotypes. Seeds were grown on MS media (A) or the media supplemented with 200 mM NaCl (B) or 200 mM NaCl + 1 µM fluridone (C) for 14 days. **(D-F)** Seed germination rate. The seed growth conditions are shown in (A-C) and the germination rates were calculated by dividing the number of germinated seeds by the total number of seeds. **(G)** Quantification of seedling phenotypes. Seeds were grown on agar plates supplemented with 200 mM NaCl and 200 mM NaCl + 1 µM fluridone for 14 days, and then the seedling phenotypes were quantified. The values indicate the means ± SD of four biological repeats, each with approximately 45 seeds.

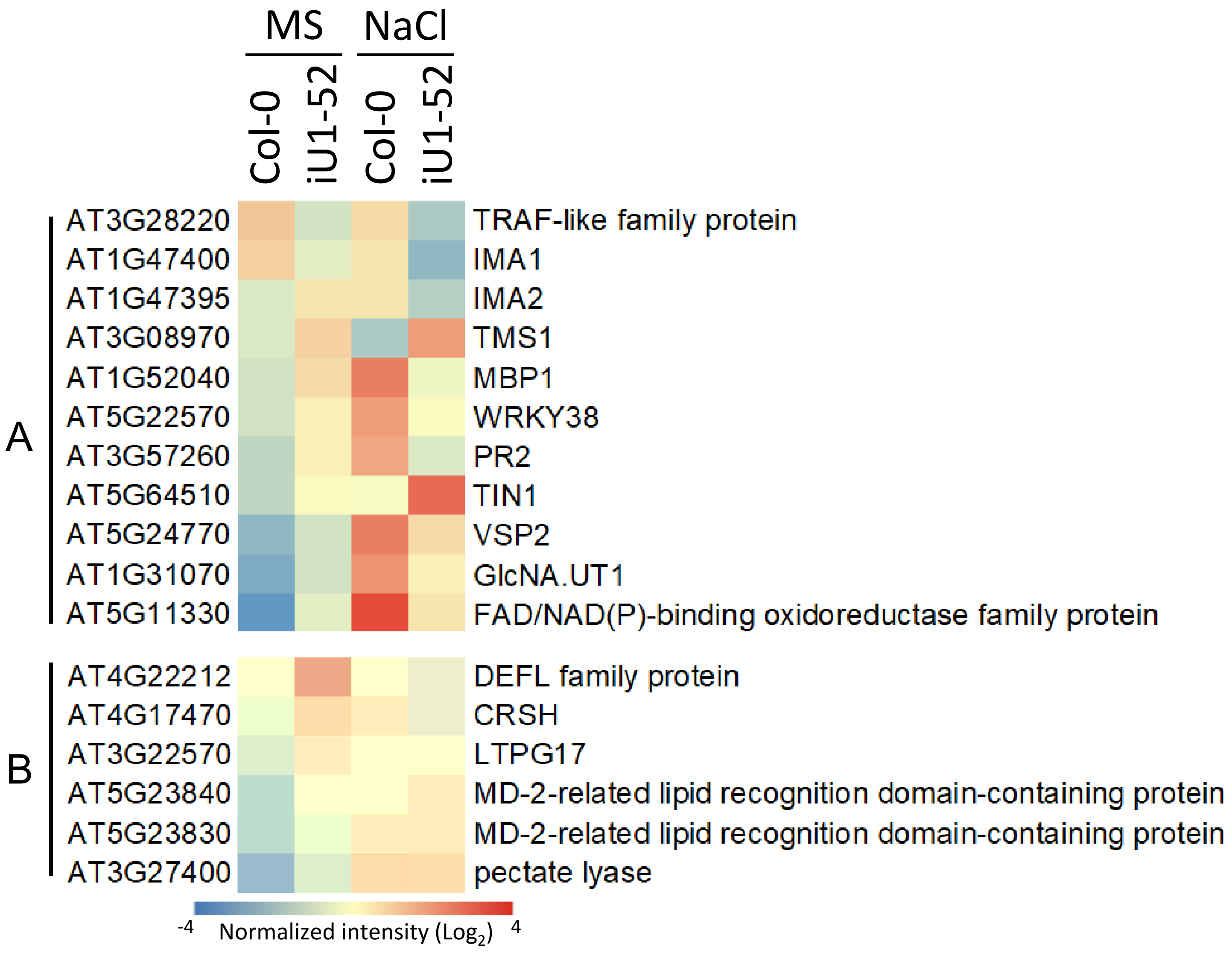

Supplementary Figure S8. Heatmap representing differentially expressed gene (DEG) profiles. **(A)** Heatmap representing the overlap of DEG between salt-free and salt-treated seedlings. **(B)** Heatmap represents DEGs only found in MS-grown seedlings.

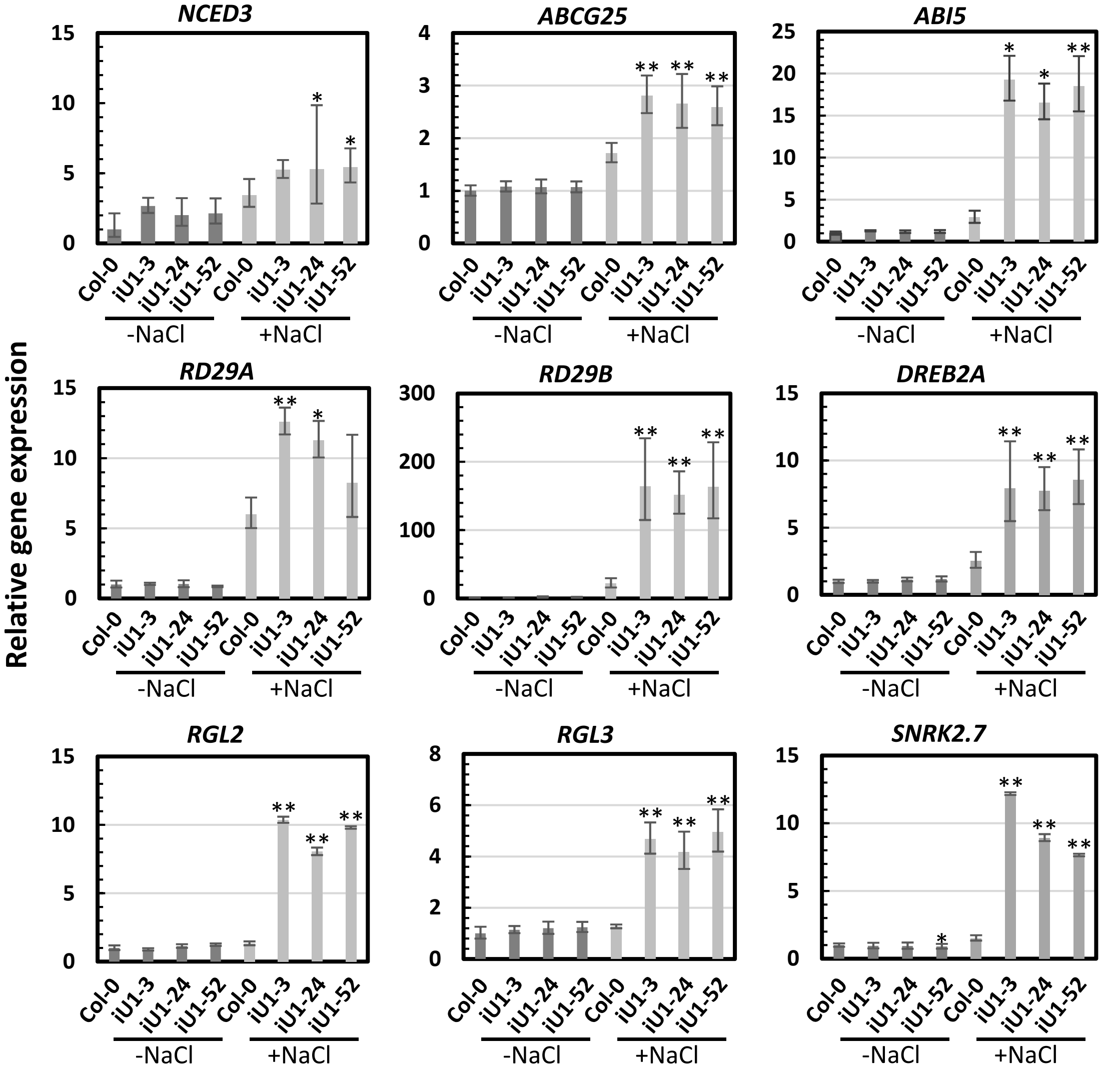

Supplementary Figure S9. Validation of microarray data by RT-qPCR. Nine DEGs shown in Figure 8D were verified by RT-qPCR. Seedlings that were grown on agar plates supplemented with or without NaCl (200 mM NaCl) for 14 days were used for RNA extraction, followed by RT-qPCR analysis. Data are normalized to *PP2A* and presented as the means ± SD of four biological replicates, each with technical triplicates. *, $p<0.05$, Student's *t*-test.

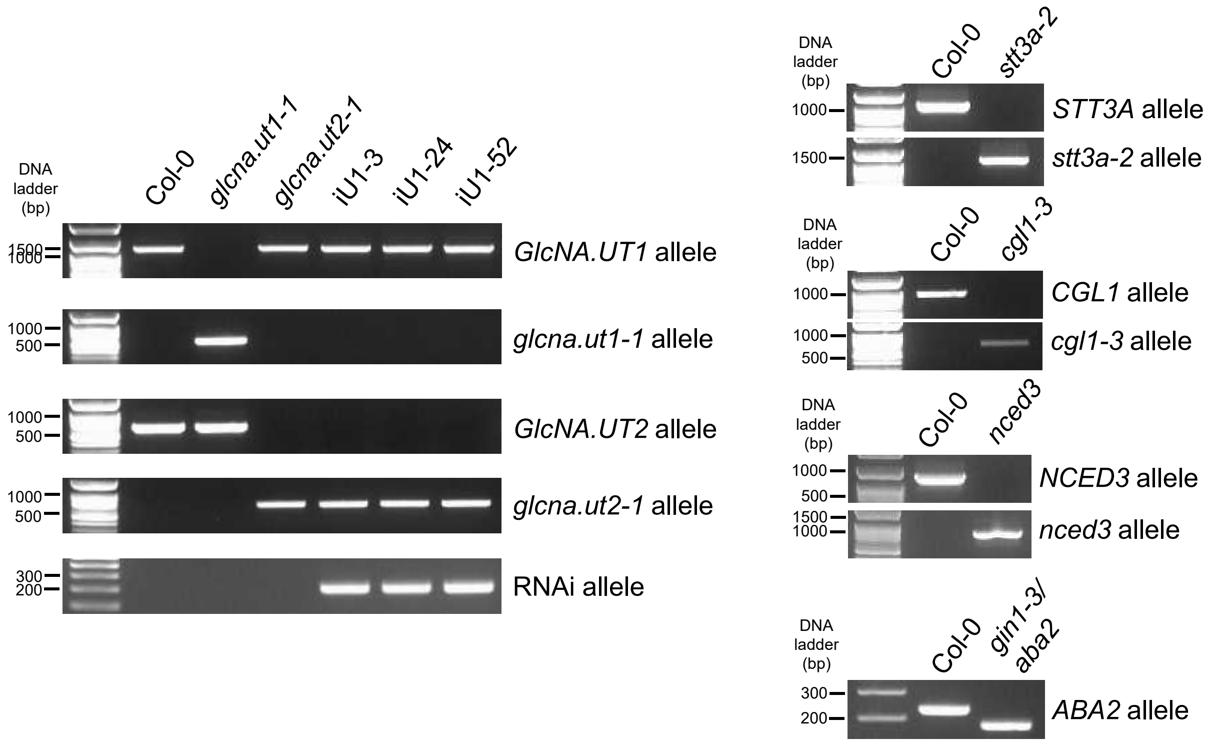

Supplementary Figure S10. Genotyping of mutants used in this study. DNA samples were obtained from the seedlings grown on 1% Suc-containing agar plates for 14 days. Primers used for genomic DNA PCR were listed in Supplementary Table S3. The mutants were requested from the ABRC stock center (OH, US) as listed in Supplementary Table S1. The *gin1-3*/*aba2* mutant shows a 53-bp deletion at the beginning of the 2nd exon and generates an early translation stop after 32 amino acids (Cheng et al., 2002).
