# Supplementary material for: Interference of Arabidopsis N-Acetylglucosamine-1-P Uridylyltransferase Expression Impairs Protein N-Glycosylation and Induces ABA-Mediated Salt Sensitivity During Seed Germination and Early Seedling Development
Source: Front Plant Sci. 2022 Jun 7;13:903272. doi: 10.3389/fpls.2022.903272 (PMC9210984; doi:10.3389/fpls.2022.903272)
Supplement: Supplementary file 2 [file Data_Sheet_2.docx]

| **Supplementary Table S1.** Arabidopsis mutant lines used in this study | | | |
| --- | --- | --- | --- |
| Allele name | Stock number | AGI number | References |
| *glcna.ut1-1* | SALK_068977 | AT1G31070 | (Chen et al., 2014) |
| *glcna.ut2-1* | SALK_150383 | AT2G35020 | (Chen et al., 2014) |
| *stt3a-2* | SALK_058814 | AT5G19690 | (Koiwa et al., 2003) |
| *cgl1-3* | SALK_073650 | AT4G38240 | (Kang et al., 2008) |
| *nced3* | GABI_129B08 | AT3G14440 | (Wan and Li, 2006) |
| *aba2*/*gin1-3* |  | AT1G52340 | (Cheng et al., 2002) |

The mutants were requested from the Arabidopsis Biological Research Center (ABRC, OH).

| **Supplementary Table S2.** Primers used for RT-qPCR | | | | |
| --- | --- | --- | --- | --- |
| AGI number | Gene name | Forward primer (5'-> 3') | Reverse primer (5'-> 3') |  |
| AT1G31070 | *GlcNA.UT1* | TATGCTCCTTCAACCGCACTT | GGTGCAAACTCCTCTTCCCTTA |  |
| AT2G35020 | *GlcNA.UT2* | TGCTTTCCTTATGCTCCTTCGA | GCAAACTCTTCCTCCCTCAACA |  |
| AT5G05320 | *UBQ10* | AGAAGTTCAATGTTTCGTTTCATGTAA | GAACGGAAACATAGTAGAACACTTATTCA |  |
| AT5G20990 | *CNX1* | GGCGAAGAAAAGAAAGGAGGAA | GCTGTCAACAAACCGTCTTCAC |  |
| AT1G09210 | *CRT2* | ATCTGATGCTGAAGACGAACCA | TCGGAATCATCTCCTCCTTCAT |  |
| AT5G42020 | *BiP2* | TCAAGGACGCTGTTGTCACTGT | GCTTGCCTTTGAGCATCATTG |  |
| AT2G47470 | *PDIL2* | TCACTCGCTCCCACATACGAA | GCATCCAAATTGGCGATGAC |  |
| AT1G13320 | *PP2A* | TAACGTGGCCAAAATGATGC | GTTCTCCACAACCGCTTGGT |  |
| AT3G14440 | *NCED3* | TCCAGATTGCTTCTGCTTCCAT | GGACCCTATCACGACGACTTCA |  |
| AT1G71960 | *ABCG25* | TCTCATGTGGTGGCACTCTGA | AGGCCTAGTCGGTCATGTACGT |  |
| AT2G36270 | *ABI5* | CTAAAACATGCATTGGCGGAGT | CAATTTCGGTTGTGCCCTTG |  |
| AT5G52310 | *RD29A* | TGCACCAGGCGTAACAGGTAA | TGCACCAGGCGTAACAGGTAA |  |
| AT5G52300 | *RD29B* | CCTGTGTCTCTGCTTTCAGCAA | CACCAGGAGCAAACGTCCTAGT |  |
| AT5G05410 | *DREB2A* | AACAGTGTTGCCAACGGTTCA | TGAGGCTTTGTAGCGGATCAA |  |
| AT3G03450 | *RGL2* | TCGTAACGGTGGTTGAGCAAG | CGAGGAAGACGATTCCGTTGT |  |
| AT5G17490 | *RGL3* | GCGGTGGAGATGGATACAGAGT | GTTTCGTTTGCCACGCAAG |  |
| AT4G40010 | *SNRK2.7* | ACGTTGCTCCGGAAGTCTTGT | CCACACATCTGCAATCTTTCCA |  |

| **Supplementary Table S3.** Primers used for genotyping | | | | |
| --- | --- | --- | --- | --- |
| Allele name | Right Primer (5'->3') | Left Primer (5'-> 3') | T-DNA Border Primer (5'-> 3') |  |
| *GlcNA.UT1* | AGAGATATTGAGGTTCGTGATTTC | GCCTTGTTCCCTACATTAGTTTC |  |  |
| *glcna.ut1-1* | AGAGATATTGAGGTTCGTGATTTC |  | ATTTTGCCGATTTCGGAAC |  |
| *GlcNA.UT2* | GTAAGCACACCTGAAAAGTAAAC | CTGGTTAGCTCTCTCAGTTTCAAG |  |  |
| *glcna.ut2-1* | GTAAGCACACCTGAAAAGTAAAC |  | ATTTTGCCGATTTCGGAAC |  |
| *RNAi* | GTTGAGAGATTGAAAGATTATGGA | GATGTGATATCTCCACTGACG |  |  |
| *NCED3* | ACAGAGGCTCTCCTCCGTAAC | GTCAGCCACGAGAAGCTACAC |  |  |
| *nced3* | ACAGAGGCTCTCCTCCGTAAC |  | ATATTGACCATCATACTCATTGC |  |
| *STT3A* | TTGTTGTGTCTATCGGCATGTAC | GGAAGTGTTGTCCTCTGCCTAA |  |  |
| *stt3a-2* | TTGTTGTGTCTATCGGCATGTAC |  | ATTTTGCCGATTTCGGAAC |  |
| *CGL1* | CATGTTCACCAAAATTGTATGTTCTA | GGCGAACTGACTGCGTACTACAAG |  |  |
| *cgl1-3* | CATGTTCACCAAAATTGTATGTTCTA |  | ATTTTGCCGATTTCGGAAC |  |
| *ABA* | AGGAGACTATGGTGCGATTGG | TGGACTCACCACGAAGCAGAC |  |  |
